# Prognosis and Immunotherapy Response With a Novel Golgi Apparatus Signature-Based Formula in Lung Adenocarcinoma

**DOI:** 10.3389/fcell.2021.817085

**Published:** 2022-01-20

**Authors:** Yupeng Jiang, Wenhao Ouyang, Chenzi Zhang, Yunfang Yu, Herui Yao

**Affiliations:** ^1^ Guangdong Provincial Key Laboratory of Malignant Tumor Epigenetics and Gene Regulation, Department of Medical Oncology, Center of Phase I Clinical Trial, Center of Breast Tumor, Sun Yat-Sen Memorial Hospital, Sun Yat-Sen University, Guangzhou, China; ^2^ Department of Hematology, Xiangya Hospital, Central South University, Changsha Hunan, China; ^3^ Artificial Intelligence & Digital Media Concentration Program, Beijing Normal University-Hong Kong Baptist University United International College, Zhuhai, China

**Keywords:** Golgi apparatus, gene signature, lung adenocarcinoma, machine algorithm, immunotherapy, anticancer drugs

## Abstract

The Golgi apparatus (GA) is a cellular organelle that participates in the packaging, modification, and transport of proteins and lipids from the endoplasmic reticulum to be further fabricated before being presented to other cellular components. Recent studies have demonstrated that GA facilitates numerous cellular processes in cancer development. Therefore, this study aimed to establish a novel lung adenocarcinoma (LUAD) risk evaluation model based on GA gene signatures. In this study, we used TCGA-LUAD (*n* = 500) as the training cohort and GSE50081 (*n* = 127), GSE68465 (442), and GSE72094 (398) as the validation cohorts. Two immunotherapy datasets (GSE135222 and GSE126044) were also obtained from a previous study. Based on machine algorithms and bioinformatics methods, a GA gene-related risk score (GARS) was established. We found that the GARS independently predicted the prognosis of LUAD patients and remained effective across stages IA to IIIA. Then, we identified that the GARS was highly correlated with mutations in P53 and TTN. Further, this study identified that GARS is related to multiple immune microenvironmental characteristics. Furthermore, we investigated GSE135222 and GSE126044 and found that a lower GARS may be indicative of an improved therapeutic effect of PD-1/PD-L1 therapy. We also found that high GARS may lead to a better response to multiple anticancer drugs. Finally, we established a nomogram to better guide clinical application. To our knowledge, this is the first study to demonstrate a novel GA signature-based risk score formula to predict clinical prognosis and guide the treatment of LUAD patients.

## Introduction

Lung adenocarcinoma (LUAD) is the predominant subtype of non-small cell lung cancer (NSCLC) (Sung et al., 2021). With the application of novel targeted drugs and immune therapy, the mortality of LUAD has slightly declined; however, the prognosis of LUAD remains unsatisfactory (Siegel et al., 2018; Cui et al., 2021). Moreover, because of tumor heterogeneity, establishing a systematic and precise method to stratify the risk of LUAD is critical.

The Golgi apparatus (GA) is a cellular organelle that participates in the packaging, modification, and transport of proteins and lipids from the endoplasmic reticulum to be further fabricated before being presented to other cellular components (Liu et al., 2021a). In addition to its well-known roles, recent studies have demonstrated that the GA facilitates numerous cellular processes in cancer development, including innate immune reactions, angiogenesis, tumor migration, and invasion (Li et al., 2019; Tao et al., 2020). Hence, some researchers have achieved satisfactory results in the treatment of tumors using manipulated Golgi-targeted nano drug delivery systems (Zhang et al., 2022). Moreover, the membranous nets that link the GA with the endoplasmic reticulum, mitochondria, autophagosomes, and endosomes provide feasible access to cellular signal transmission and subsequent effector responses (Li et al., 2019; Tao et al., 2020). With the in-depth study of cellular organelles, scientists have gradually focused on the role of the GA in the development and progression of cancer. So far, there has been no relevant research on the prognostic significance of GA-related gene set-based formulas in cancer. Thus, investigating GA-related gene sets in LUAD may provide valuable therapeutic guidance in analyzing the most relevant function of the GA related to tumor prognosis.

In the present study, we aimed to construct a GA signature-based LUAD prognosis forecasting system using bioinformatics to guide further clinical treatment.

## Materials and Methods

### Data Acquisition

Gene expression data, gene mutation information, and clinical survival data of LUAD patients were acquired from The Cancer Genome Atlas (TCGA) database (TCGA-LUAD) (Schabath et al., 2016) and the Gene Expression Omnibus (GEO) database (GSE50081, GSE68465, GSE72094) (Zhang et al., 2021; Zhou et al., 2021). TCGA-LUAD was used as the training cohort, while GSE50081, GSE68465, and GSE72094 were used as the validation cohorts. The “sva” package of the R software was adopted to rectify the batch effect between different datasets using the “combat” algorithm. GA-related genes were extracted from the hallmark gene set in the Molecular Signatures Database v7.0, which includes 1,608 GA-related genes. This study was approved by the Ethics and Research Committees of Sun Yat-Sen Memorial Hospital, Sun Yat-Sen University.

### Screening of Differentially Expressed Golgi Apparatus-Related Genes

Information on the expression of 1,608 GA-related genes was acquired from TCGA-LUAD database. Differentially expressed genes (DEGs) between normal lung tissue and LUAD were then identified through the Wilcoxon test according to Log2Fc <−1 or >1 and *p* < 0.05, with Log2Fc > 1 and Log2Fc < −1 indicating upregulated and downregulated genes, respectively. Heatmaps were generated to express differential genes.

### Functional Exploration of Differentially Expressed Genes

R software package (clusterprofiler, version 3.12) was used to perform Gene Ontology (GO) and Kyoto Encyclopedia of Genes and Genomes (KEGG) pathway enrichment analysis. With the use of Fisher’s exact test, those with false discovery rate (FDR)-corrected *p*-values of less than 0.05 were regarded as marked indicators.

### Construction and Verification of the Golgi Apparatus Gene-Related Risk Score Formula

First, RNA expression in TCGA-LUAD, GSE50081, GSE68465, and GSE72094 datasets was cross-checked to identify co-expressed and differentially expressed GA-related genes. Consequently, univariate Cox analysis of overall survival (OS) was applied to screen for GA-related genes with prognostic value. Subsequently, least absolute shrinkage and selection operator (LASSO) regression with 10-fold cross-validation was performed, 1,000 cycles of the “glmnet” package were run using the R software, and 1,000 random stimulations were set. Based on the best lambda value, the optimal possible gene was selected to construct the model, and a GA gene-related risk score (GARS) was constructed.

The GARS was calculated based on the expression of each gene and its corresponding regression coefficients based on the following equation: risk score = ∑ genes Cox coefficient × gene expression. The patients were then categorized into the high and low GARS groups according to the best cutoff value, using the R package of “surv_cutpoint” in “survminer.” The predictive sensitivity of the GARS was painted via the R Package “survivalROC” for estimation. The model effectiveness was evaluated in the validation set using the same coefficient and cutoff values that were used in the training set.

Given the lack of information on immune therapy in TCGA-LUAD cohort, the immune therapy predictive capability of the risk score formula was evaluated using the two lung cancer cohorts GSE135222 and GSE126044 (Cho et al., 2020; J. Y.; Kim et al., 2020).

### Establishment of the Nomogram

This study used the Cox regression model along with the R package “rms” to build an OS prediction nomogram that set 1-, 3-, and 5-year OS as the endpoints. The C-index was used to estimate the discriminative ability of the nomogram. Calibration plots were used to visualize the consistency between the predicted and factual 1-, 3-, and 5-year OS.

### Differential Analysis of Immune Cell Infiltration, Immune Function, Immune Checkpoint, and Validation of Immunotherapy Response

Immune cell infiltration was identified using timer 2.0 (cistrome.shinyapps.io/timer/) via the MCPCOUNTER, CIBERSORT, QUANTISEQ, Timer, CIBERSORT-ABS, EPIC, and XCELL algorithms. The “gsva” R package was applied to study the single-sample gene set enrichment analysis (ssGSEA) to count the activities of 13 immune pathways. Immune checkpoint-related genes were retrieved as previously described (Dib et al., 2017). The ESTIMATE algorithm was used to calculate the stromal and immune scores, as well as the ESTIMATE score of TCGA-LUAD samples.

### Predicting Drug Therapeutic Response

To predict the immunotherapy sensitivity, the Immunophenoscore (IPS) was calculated using the Cancer Immunome Atlas (https://tcia.at/). The IC_50_ of common chemotherapeutic drugs was counted in the training group via the administered “pRRophetic” package to evaluate the predictive capability of the GARS for drug therapeutic responses. The Wilcoxon rank test was then used to compare the difference in IC_50_ between the low and high GARS groups. Finally, we applied the “ggplot” R package to paint the result in the histogram.

### Statistical Analysis

DEGs were screened using the Wilcoxon test and compared using Fisher’s exact test. Univariate Cox analysis of OS was performed to identify relevant genes and their prognostic value. Kaplan–Meier survival curves were generated and compared between the two groups using the log-rank test. The association between the prognostic model risk score and immune score was assessed using Spearman’s correlation analysis. All statistical analyses were performed using R version 4.0.0 (R-project.org) and its adequate packages. Statistical significance was set at *p* ≤ 0.05.

## Result

**FIGURE 1:**
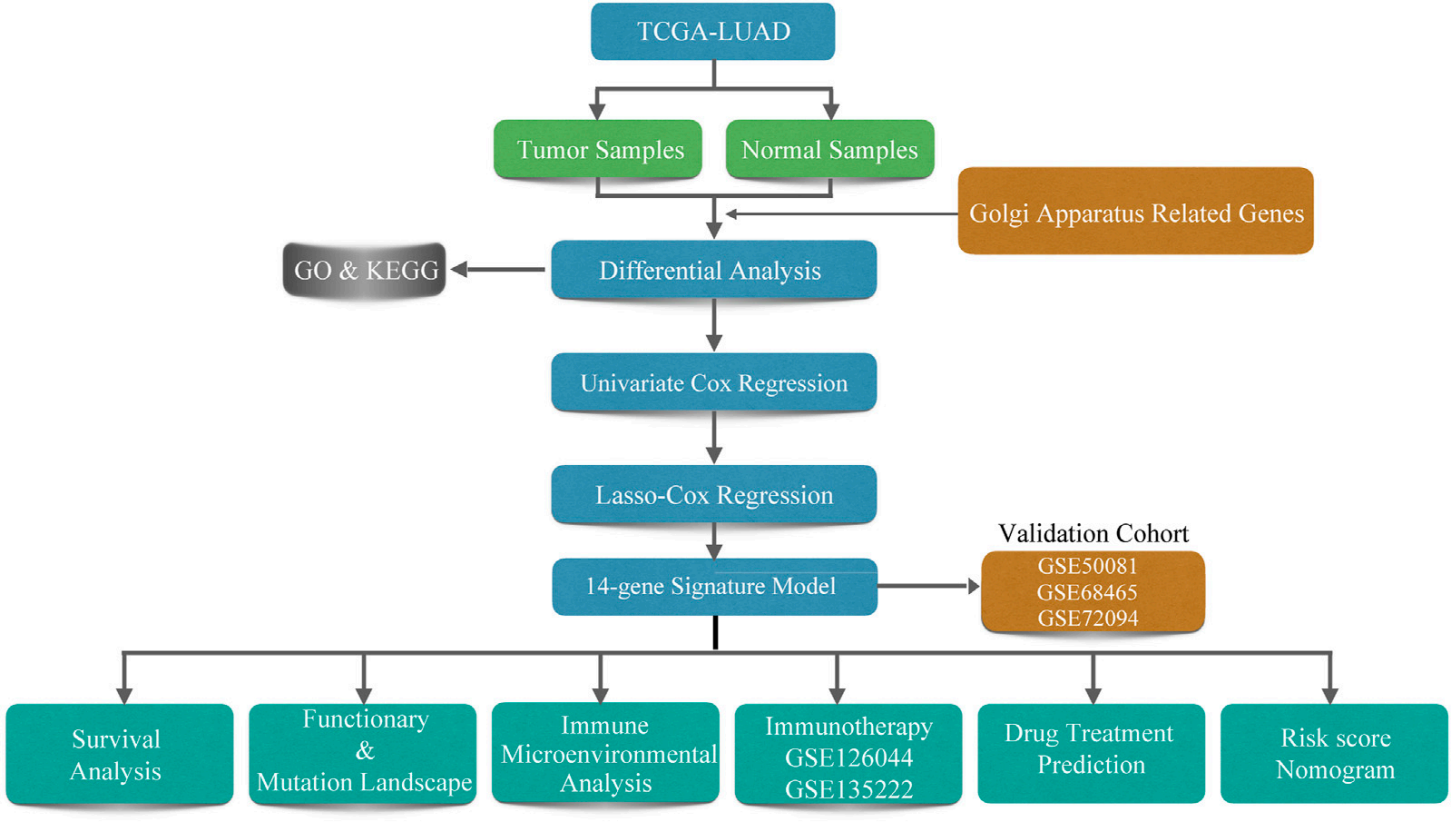
Flowchart of the study.

A total of 500 LUAD patients from the training sets and 967 LUAD patients from the validation sets were selected. The detailed clinical features of these patients are summarized in Table 1. A flowchart of the study is presented in Figure 1.

**TABLE 1:** | Clinical information of patients with LUAD in this study.

Corhort	TCGA-LUAD	GSE50081	GSE72094	GSE68465
Number of patients	*n* = 500	*n* = 127	*n* = 398	*n* = 442
Age (mean ± SD)	65.26 ± 10.05	65.76 ± 10.29	69.36 ± 9.45	64.39 ± 10.09
Follow up time (mean ± SD) (days)	907.93 ± 896.64	1,510.77 ± 964.23	791.91 ± 402.69	1,600.05 ± 1,102.46
Follow up status				
Alive	318 (63.6%)	76 (59.84%)	285 (71.61%)	206 (46.61%)
Dead	182 (36.4%)	51 (40.16%)	113 (28.39%)	236 (53.39%)
Gender				
Male	230 (46%)	65 (51.18%)	176 (44.22%)	223 (50.45%)
Female	270 (54%)	62 (48.82%)	222 (55.78%)	219 (49.55%)
Clinical stage				
Stage I	268 (53.6%)	92 (72.44%)	254 (63.82%)	—
Stage II	119 (23.8%)	35 (27.56%)	67 (16.83%)	—
Stage III	80 (16%)	—	57 (14.32%)	—
Stage IV	25 (5%)	—	15 (3.77%)	—
Unknown	8 (1.6%)	—	5 (1.26%)	—
T stage				
T1	167 (33.4%)	43 (33.86%)	—	—
T2	267 (53.4%)	82 (64.57%)	—	—
T3a	45 (9%)	2 (1.57%)	—	—
T4	18 (3.6%)	18 (3.6%)	—	—
Unknown	3 (0.6%)	3 (0.6%)	—	—
M stage				
M0	332 (66.4%)	127 (100%)	—	—
M1	24 (4.8%)	—	—	—
Unknown	144 (28.8%)	—	—	—
N stage				
N0	324 (64.8%)	94 (74.02%)	—	—
N1	94 (18.8%)	33 (25.98%)	—	—
N2	69 (13.8%)	—	—	—
N3	2 (0.4%)	—	—	—
Unknown	11 (2.2%)	—	—	—

Note. LUAD, lung adenocarcinoma.

### Differentially Expressed and Golgi Apparatus-Related Genes

**FIGURE 2:**
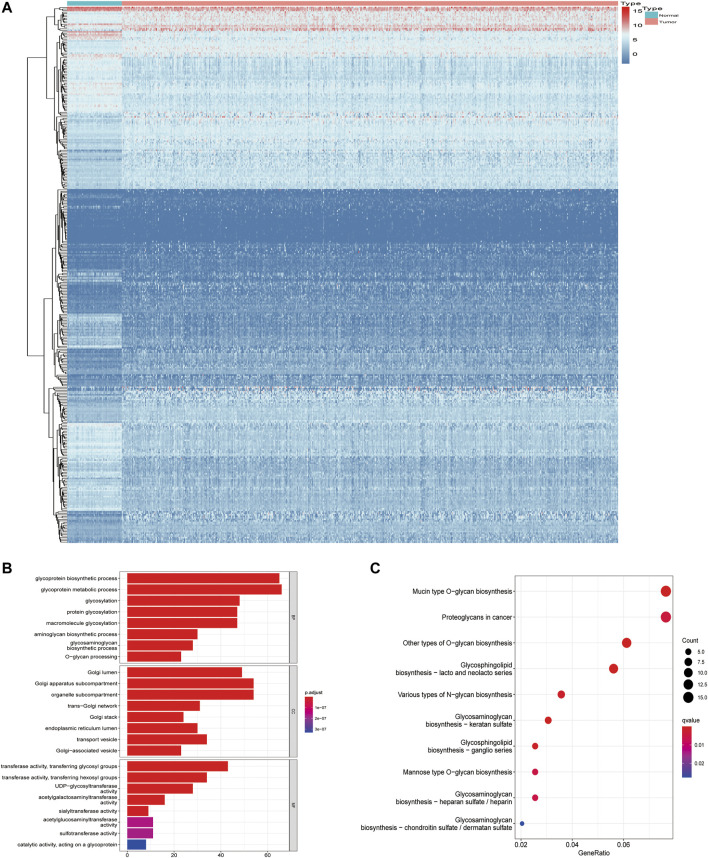
Screening of differentially expressed Golgi apparatus-related genes. **(A)** Heatmaps of differentially expressed Golgi apparatus-related mRNAs. **(B)** GO enrichment of Golgi apparatus-related DEGs. **(C)** KEGG pathways of Golgi apparatus-related DEGs. GO, Gene Ontology; DEGs, differentially expressed genes; KEGG, Kyoto Encyclopedia of Genes and Genomes.

In the training set, by applying the Wilcoxon test according to Log2Fc < −1 or >1 and *p* < 0.05, we identified 353/1,608 GA-related DEGs between LUAD and adjacent normal tissues. With Log2Fc > 1 and Log2Fc < −1 indicating upregulated and downregulated genes, respectively, we found 212 upregulated and 141 downregulated GA-related genes (Figure 2A).

### Functional Analysis of Golgi Apparatus-Related Differentially Expressed Genes

Through GO enrichment analysis, we identified the most obvious GO category with significant enrichment of GA-related genes. The most significantly altered GA-related genes were involved in glycoprotein metabolic processes (Figure 2B; Supplementary Table S1A). Subsequently, we performed KEGG analysis and identified the top KEGG category with significant enrichment of GA-related genes. The altered GA-related genes were mostly associated with mucin-type *O*-glycan biosynthesis (Figure 2C; Supplementary Table S1B).

### Predictive Capability of the Golgi Apparatus Gene-Related Risk Score

**FIGURE 3:**
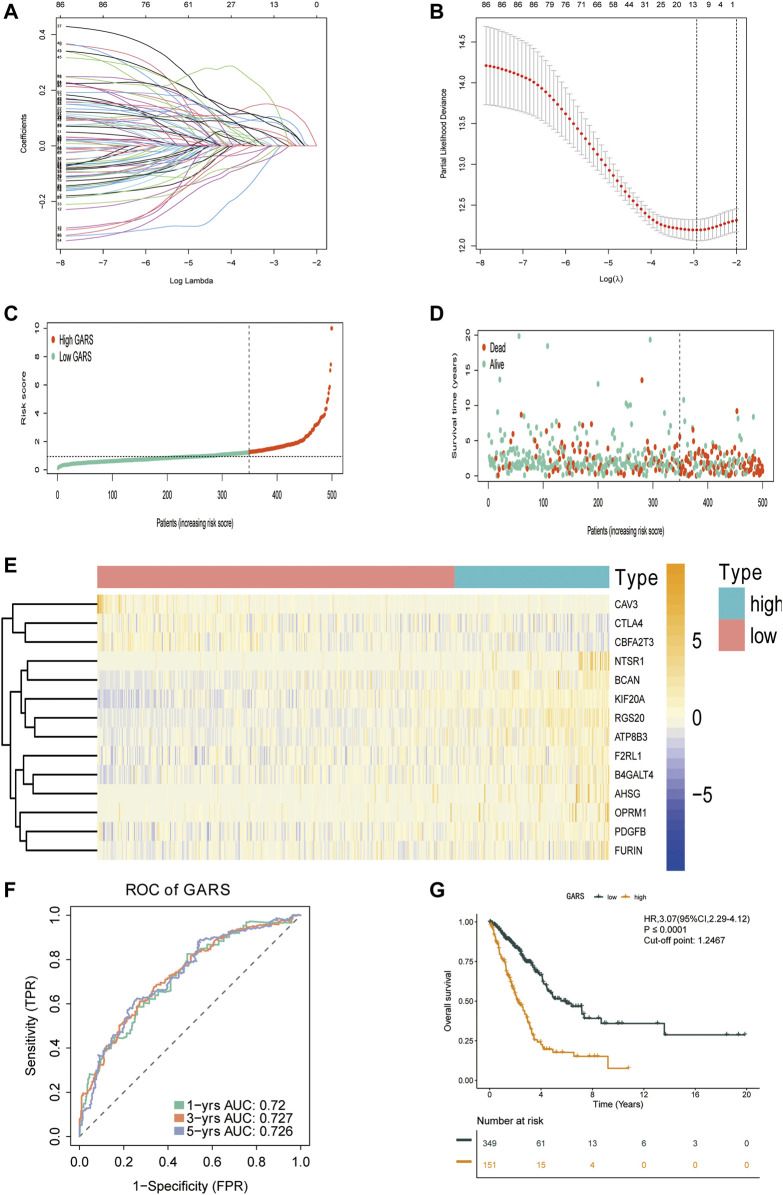
Construction of the GARS formula. **(A)** LASSO coefficient profile plots of the 86 prognostic-related genes showing that the variations in the size of the coefficients of parameters shrink with an increasing value of the k penalty. **(B)** Penalty plot for the LASSO model for the 86 prognostic genes with error bars denoting the standard errors. **(C)** Distribution of GARS for the training set. **(D)** Patterns of the survival time and survival status between the high GARS and low GARS groups for the training set. **(E)** Heatmaps of the 14 prognostic genes for each patient in the training set. **(F)** Time-related ROC analysis exhibited the prognostic value of the GARS in the training set. **(G)** Kaplan–Meier survival curve of the patients in the high GARS and low GARS groups for OS in the training set. GARS, Golgi apparatus gene-related risk score; LASSO, least absolute shrinkage and selection operator; ROC, receiver operating characteristic; OS, overall survival.

A total of 11,047 genes were co-expressed, among which 242 of 353 GA-related DEGs were selected. These 242 genes were then used in univariate Cox regression analysis. A total of 86 prognostic genes were identified. To avoid overfitting the prognostic model, LASSO regression analysis was performed (Figures 3A,B). Finally, 14 genes were selected and included in the risk score formula: GARS = RGS20 × 0.181 − B4GALT4 × 0.033 + PDGFB × 0.179 + NTSR1 × 0.117 + ATP8B3 × 0.044 + AHSG × 0.082 − CTLA4 × 0.142 + KIF20A × 0.159 + BCAN × 0.133 − 0.321 × CAV3 − CBFA2T3 × 0.109 + OPRM1 × 0.328 + FURIN × 0.102 + F2RL1 × 0.120. Based on the optimistic cutoff, 151 and 349 patients were categorized into the high GARS and low GARS groups, respectively (Figures 3C–E). Kaplan–Meier survival analysis showed a lower OS in the high GARS group than in the low GARS group (hazard ratio (HR) = 3.07; 95% CI 2.29–4.12; *p* < 0.0001). The area under the curve (AUC) for predicting 1-, 3-, and 5-year OS was 0.720, 0.727, and 0.726, respectively (Figures 3F,G). These results showed that the risk model based on the 14 genes had high accuracy in predicting the OS of patients with LUAD.

### Stability of the Golgi Apparatus Gene-Related Risk Score Formula

**FIGURE 4:**
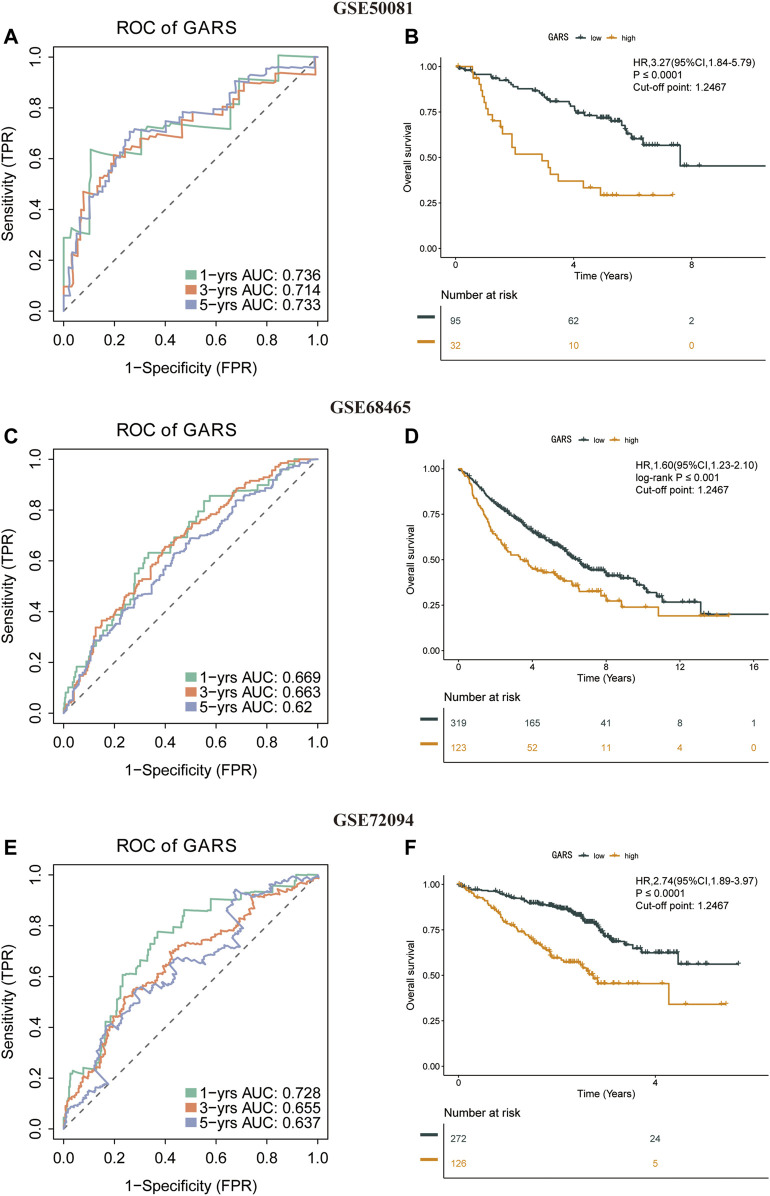
Stability of the GARS formula in the validation sets. **(A)** Time-related ROC analysis proved the prognostic value of the GARS in the GSE50081. **(B)** Kaplan–Meier survival curve of the patients in the high GARS and low GARS groups for OS in the GSE50081. **(C)** Time-related ROC analysis proved the prognostic value of the GARS in the GSE68465. **(D)** Kaplan–Meier survival curve of the patients in the high GARS and low GARS groups for OS in the GSE68465. **(E)** Time-related ROC analysis proved the prognostic value of the risk score in the GSE72094. **(F)** Kaplan–Meier survival curve of the patients in the high GARS and low GARS groups for OS in the GSE72094. GARS, Golgi apparatus gene-related risk score; ROC, receiver operating characteristic; OS, overall survival.

To check the stability of the GARS developed from the training set, patients in the validation sets (GSE50081, GSE68465, and GSE72094) were also divided into the high and low GARS groups according to the same cutoff value and risk formula as those in TCGA cohort. The results showed a markedly lower OS in the high GARS group than in the low GARS group (Figures 4B,D,F). In the GSE50081, the AUCs for predicting the 1-, 3-, and 5-year OS were 0.736, 0.714, and 0.733, respectively (HR = 3.27; 95% CI = 1.84–5.79; *p* < 0.0001) (Figure 4A). In the GSE68465 set, the AUCs for predicting the 1-, 3-, and 5-year OS were 0.669, 0.663, and 0.62, respectively (HR = 1.60; 95% CI = 1.23–2.10; *p* < 0.001) (Figure 4C). In the GSE72094 cohort, the corresponding AUCs were 0.728, 0.655, and 0.637, respectively (HR = 2.74; 95% CI = 1.89–3.97; *p* < 0.0001) (Figure 4E).

### Subgroup Analysis of Different Stages With the Golgi Apparatus Gene-Related Risk Score Formula

**FIGURE 5:**
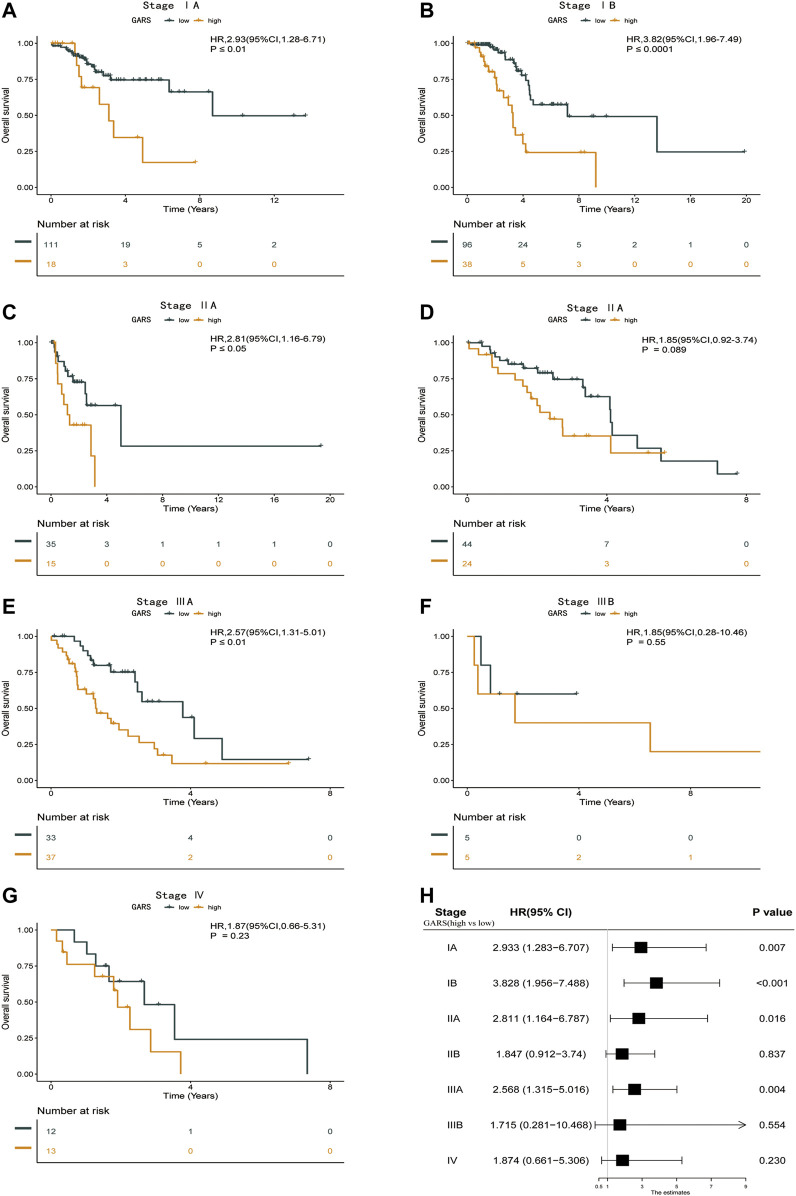
Subgroup analysis of different stages with the GARS formula. **(A)** Kaplan–Meier survival curve of the patients in the high GARS and low GARS groups for OS in stage IA LUAD patients. **(B)** Kaplan–Meier survival curve of the patients in the high GARS and low GARS groups for OS in stage IB LUAD patients. **(C)** Kaplan–Meier survival curve of the patients in the high GARS and low GARS groups for OS in stage IIA LUAD patients. **(D)** Kaplan–Meier survival curve of the patients in the high GARS and low GARS groups for OS in stage II LUAD patients. **(E)** Kaplan–Meier survival curve of the patients in the high GARS and low GARS groups for OS in stage IIIA LUAD patients. **(F)** Kaplan–Meier survival curve of the patients in the high GARS and low GARS groups for OS in stage IIIB LUAD patients. **(G)** Kaplan–Meier survival curve of the patients in the high GARS and low GARS groups for OS in stage Ⅳ LUAD patients. **(H)** Forest plot illustrating the survival of the patients in the high GARS and low GARS groups in different stages. GARS, Golgi apparatus gene-related risk score; OS, overall survival; LUAD, lung adenocarcinoma.

At present, the treatment of LUAD is usually based on the clinical stage (Bang et al., 2020). Particularly, patients with IA are not recommended for any further anticancer therapy after the removal of the tumor (Chou et al., 2021). However, the prognosis of patients at the same stage was divergent. Thus, we analyzed the association between the different stages and GARS in TCGA-LUAD database. The GARS accurately identified stage IA LUAD patients with different prognoses (HR = 2.93; 95% CI 1.28–6.71; *p* < 0.01) (Figure 5A). Further, the GARS remained effective across stages IB to IIIA (Figures 5B–E). However, it did not distinguish between the prognosis of patients with stage IIIB and stage IV LUAD (Figures 5F,G). This may be due to the low population of these groups. Besides, the result above is also illustrated in a forest plot (Figure 5H).

### Functional Analysis Between the High Golgi Apparatus Gene-Related Risk Score Group and the Low Golgi Apparatus Gene-Related Risk Score Group

**FIGURE 6:**
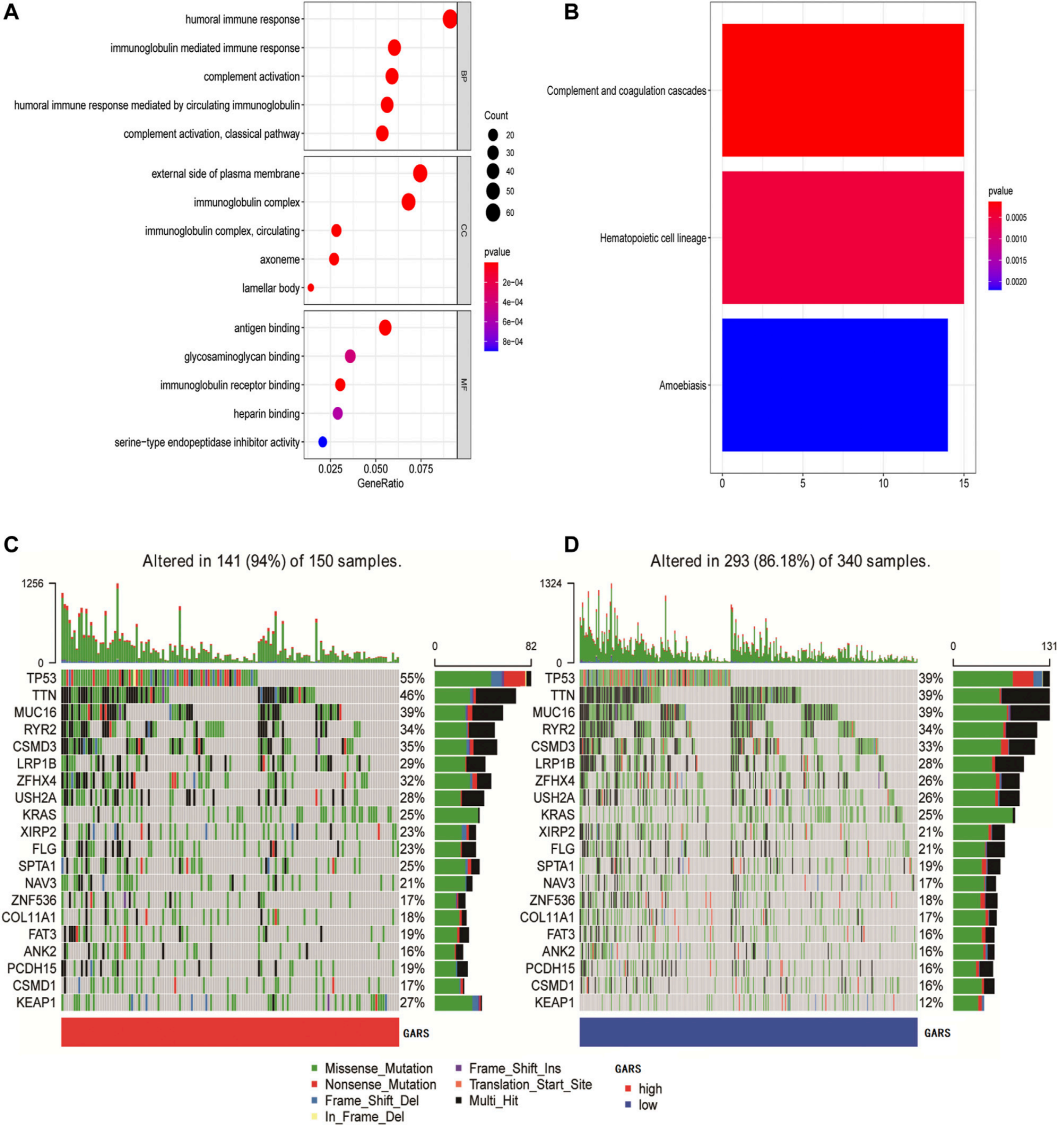
Functional analysis and identification of mutation landscape. **(A)** GO enrichment between the high GARS patients and low GARS patients in TCGA-LUAD cohort. **(B)** KEGG pathways between the high GARS patients and low GARS patients in TCGA-LUAD cohort. **(C, D)** Comparison of the mutation landscape between groups with high and low GARS. GO, Gene Ontology; GARS, Golgi apparatus gene-related risk score; KEGG, Kyoto Encyclopedia of Genes and Genomes.

Further analysis of the differences in GO enrichment pathways between the low and high GARS groups showed that the most different pathways were the humoral immune response, external side of the plasma membrane, and antigen binding (Figure 6A; Supplementary Table S2A). We also analyzed the differences in KEGG enrichment pathways between the low and high GARS groups, indicating that complement and coagulation cascades are the most significant pathways (Figure 6B; Supplementary Table S2B). This may be a potential reason for the lower OS in the high GARS group.

### Identification of the Golgi Apparatus Gene-Related Risk Score-Associated Mutation Landscape

The mutation landscapes in the high and low GARS groups were compared. The results showed that more mutation events occurred in samples with higher GA scores, with TP53 and TTN mutations being the predominant alterations in these samples (Figures 6C,D).

### Immune Status Analysis

**FIGURE 7:**
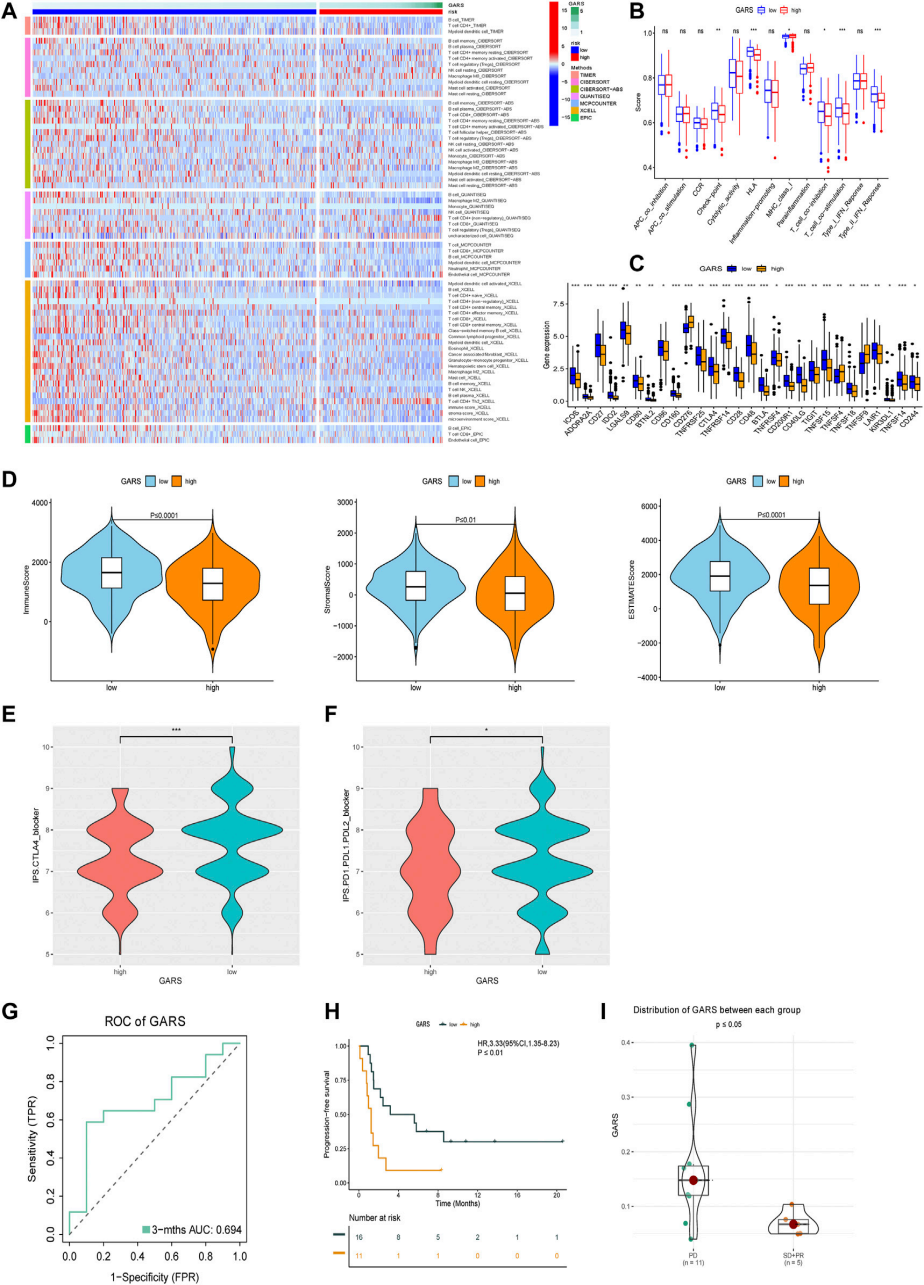
Analysis of GARS-related immune status and validation of immunotherapy response. **(A)** Heatmap for immune responses based on MCPCOUNTER, CIBERSORT, QUANTISEQ, Timer, CIBERSORT-ABS, EPIC, and XCELL algorithms among high GARS group and low GARS group. **(B)** Immune pathway analysis. **(C)** The expression of immune-related checkpoints among high and low GARS groups. **(D)** The relationship between GARS, Immune score, Stromal score, and ESTIMATE score. **(E, F)** IPS score between high GARS group and low GARS group. **(G)** ROC analysis proved the prognostic value of the GARS in the PD-1/PD-L1 treatment cohort GSE135222. **(H)** Kaplan–Meier survival curve of the patients in the high GARS and low GARS groups for OS in the PD-1/PD-L1 treatment cohort GSE135222. **(I)** Analysis of the immunotherapy response between high and low GARS group in the PD-1/PD-L1 treatment cohort GSE126044. GARS, Golgi apparatus gene-related risk score; IPS, Immunophenoscore; ROC, receiver operating characteristic; OS, overall survival.

The relationship between the GARS and immune status of the patients in TCGA cohort was investigated. There were significant alterations in immune cells (Figure 7A). The immune-related pathway also significantly differed, including CCR, HLA, T CELL co-inhibition, T-cell co-stimulation, and type II IFN response. Subsequently, we compared the expression of immune checkpoint-related genes between the high GARS and low GARS groups. In this study, we found that ICOS, ADORA2A, CD27 IDO2, LGALS9, CD80, BTNL2, BD86, CD160, TNFRSF25, CTLA4, TNFRSF14, CD28, CD48, BTLA, TNFRSF4, CD200R1, CD40LG, TIGHT, TNFSF15, TNFSF18, KIR3DL1, TNFSF14, and CD244 were significantly lower in the high GARS group (Figure 7C). The tumor immune microenvironment was also assessed using the immune score, ESTIMATE score, and stromal score, which also illustrated the negative correlation between GARS and immune reaction (Figure 7D). These results indicated stronger tumor immune activities in the low GARS group than in the high GARS group.

### Immune Treatment Analysis

Because of the high immune connection between GARS and immune status, we further estimated the prognostic predictive value of GARS with immune therapy. Surprisingly, GARS not only correlated with the IPS score of PD-1 and CTLA4 but also correlated with the prognosis of lung cancer patients who received PD-1/PD-L1 therapy (Figures 7E–I).

### Comparison of the Sensitivity to Anti-Lung Cancer Drugs Between Patients With Different Golgi Apparatus Gene-Related Risk Score

**FIGURE 8:**
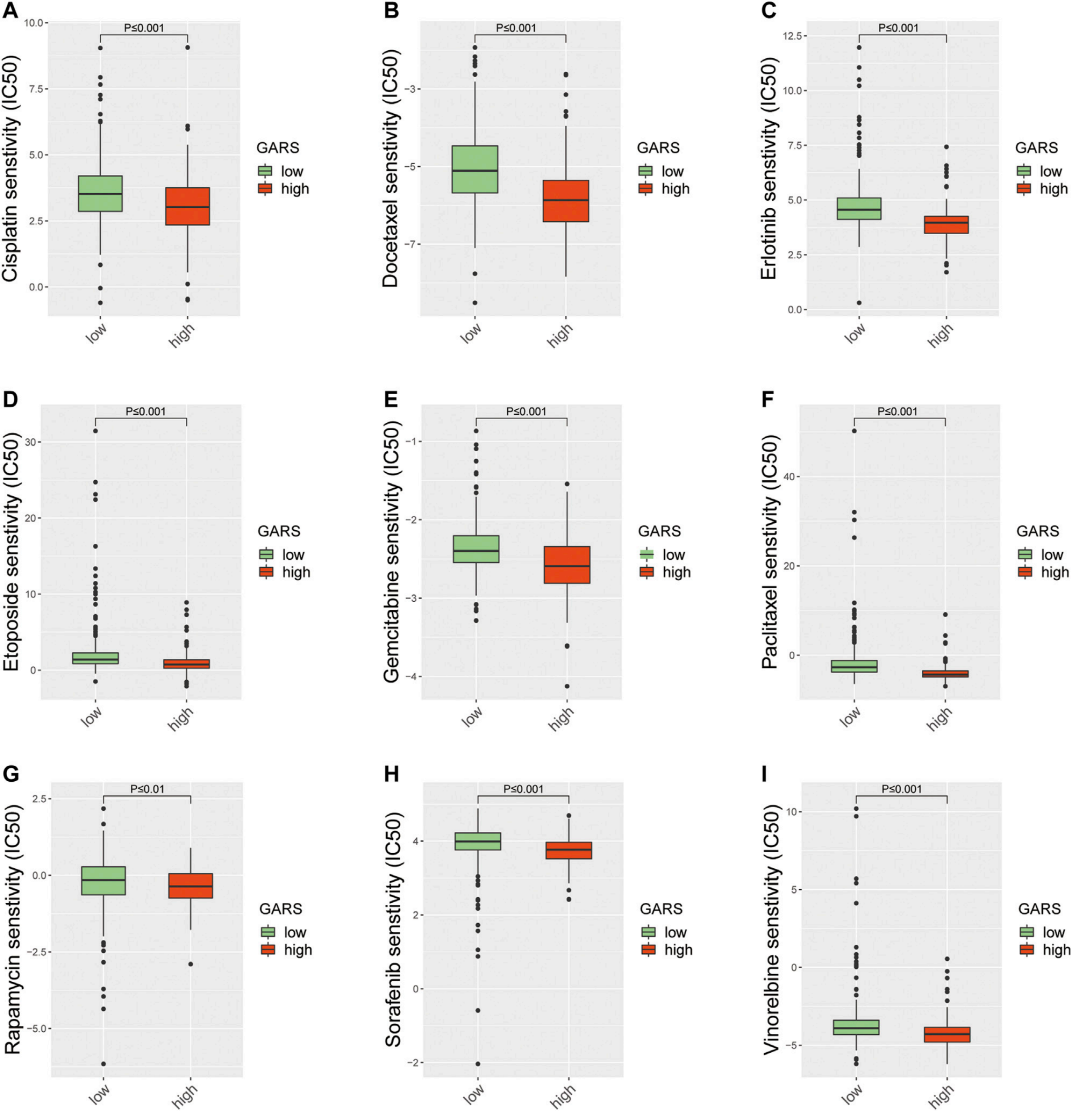
The difference of anticancer drug sensitivity between the high GARS group and low GARS group. **(A)** The IC_50_ of cisplatin among groups. **(B)** The IC_50_ of docetaxel among groups. **(C)** The IC_50_ of erlotinib among groups. **(D)** The IC_50_ of etoposide among groups. **(E)** The IC_50_ of gemcitabine among groups. **(F)** The IC_50_ of paclitaxel among groups. **(G)** The IC_50_ of rapamycin among groups. **(H)** The IC_50_ of sorafenib among groups. **(I)** The IC_50_ of vinorelbine among groups. GARS, Golgi apparatus gene-related risk score.

Different molecular subtypes of LUAD should guide clinical decisions. Therefore, the sensitivity to nine common anti-LUAD drugs was compared between the high and low GARS groups to determine potential treatment modalities. The results demonstrated that the IC_50_s of cisplatin, docetaxel, erlotinib, etoposide, gemcitabine, paclitaxel, rapamycin, sorafenib, and vinorelbine were significantly higher in patients with higher GARS (Figures 8A–I). These drugs include almost all the common drugs used to treat LUAD patients according to the National Comprehensive Cancer Network (NCCN; https://www.nccn.org) guidelines for NSCLC. This fully shows the value of the GARS.

### Golgi Apparatus Gene-Related Risk Score Independently Predict the Overall Survival of Lung Adenocarcinoma

In univariate analysis according to OS, GARS and stage were identified as significant prognostic factors in predicting OS (Supplementary Figure S1A). After other potential confounding factors were controlled, GARS and stage were found to be independent prognostic factors (Supplementary Figure S1B). These results also indicate that GARS may help to further stratify the prognosis of patients at different stages.

### The Construction and Validation of the Golgi Apparatus Gene-Related Risk Score-Related Nomogram

**FIGURE 9:**
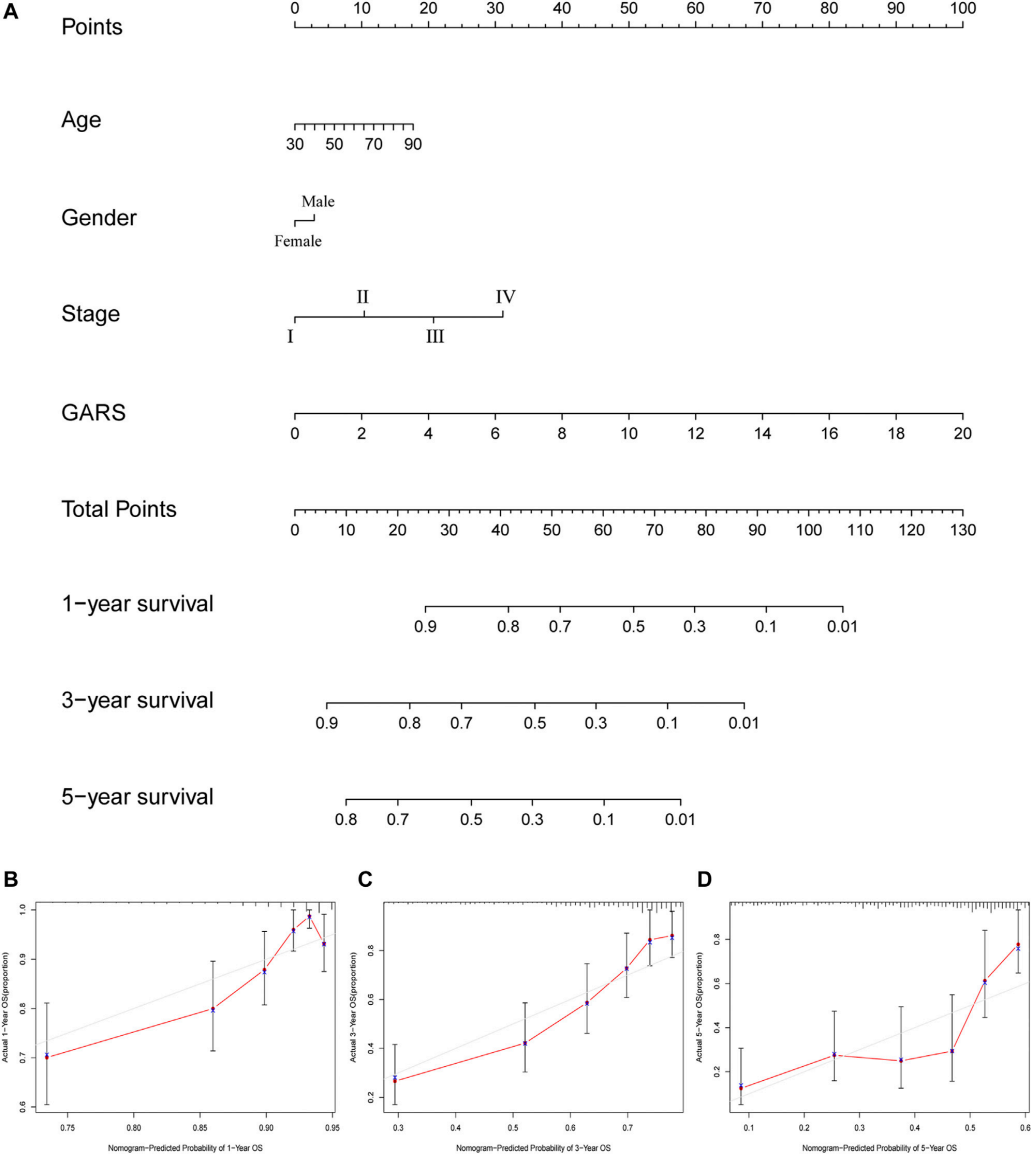
The calculation of GARS-related prognostic nomogram. **(A)** The prognostic nomogram to predict the 1-, 3-, and 5-year OS of LUAD patients. For each patient, we calculated the points of the clinical–pathological features and summed up the points to obtain the total points. The predicted 1-, 3-, and 5-year OS can be estimated based on the total points of each patient. **(B)** The calibration curves for predicting patient survival at 1-year OS. **(C)** The calibration curves for predicting patient survival at 3-year OS. **(D)** The calibration curves for predicting patient survival at 5-year OS. GARS, Golgi apparatus gene-related risk score; OS, overall survival; LUAD, lung adenocarcinoma.

A nomogram was established based on the noted parameters, including age, stage, sex, and GARS. As shown in the nomogram, each risk factor was associated with a specific score, and the calculated scores could accurately predict the prognosis of 1-, 3-, and 5-year survival rates (Figures 9A–D). This also provides a suitable clinical application scenario for GARS.

## Discussion

LUAD is a major type of lung cancer with a high incidence rate (Li Z. et al., 2021; Wang et al., 2021). The prognosis of LUAD remains devastating because of its obscure pathogenesis (Peng et al., 2021). Therefore, individual therapies for patients with LUAD are needed. In the past decade, the study of genomic and transcriptional factors has rapidly pushed the individualized treatment of tumors. However, most of these studies were based on cellular function, cellular structure-based investigations, which are rare. This study, to our best knowledge, is the first to use the Golgi apparatus gene set to carry out this kind of research. In this study, a novel formula, GARS, which independently forecasts the prognosis of LUAD patients, was developed and validated. This may provide a precise method to evaluate prognosis and guide treatment in LUAD patients.

The Golgi complex is a membrane-bound organelle that lies in a key position of multiple cellular pathways (Lujan and Campelo, 2021). The main functions of the Golgi apparatus are to maintain cellular protein and lipid homeostasis and to mediate protein sorting and export (Lujan and Campelo, 2021). Accumulating evidence has shown that a proper Golgi structure is essential for its suitable functions, especially glycosylation (X. Zhang, 2021). Notably, altered glycosylation of cell surface proteins and lipids is a well-accepted cancer hallmark (Martins et al., 2021; Zhang, 2021).

Because the promising potential of GA genes and cellular structure is the foundation of cellular function, this study first used the GA signature, which was extracted from the Molecular Signatures Database (MSigDB), to predict the prognosis of LUAD patients. In summary, 14 genes were included in the GARS formula for LUAD prognosis. By manipulating this formula, we classified LUAD patients into the high and low GARS groups. The formula had AUCs of 0.720, 0.727, and 0.726 for predicting 1-, 3-, and 5-year OS, respectively, indicating its high reliability and accuracy. Furthermore, the high GARS group had a significantly lower OS than the low GARS group. The risk score also showed high predictive capability in the GSE72094, GSE68465, and GSE50081 validation sets. Subsequently, to better guide clinical prognosis prediction, we applied GARS at different clinical stages. Surprisingly, we found that the risk score remained significantly effective across stages IA to IIIA. However, the risk score did not distinguish between the risk in stage IIIB and stage IV. It should be noted that stage IA lung cancer is usually treated only by surgery (Chang et al., 2021), indicating that patients with high GARS IA LUAD need further treatment.

Furthermore, among the 14 genes, TP53 and TTN were found to be mutating more in the high GARS group. Recently, mutations in these two genes were found to be correlated with the response to immunotherapy (Z. Liu et al., 2021b; Muñoz-Fontela et al., 2016). Mutation or loss of p53 in cancers can influence the activity and recruitment of T cells, which can induce immune evasion (Blagih et al., 2020). The gain-of-function p53 mutation type can induce the accumulation of neutrophils, which leads to resistance to immunotherapy (Siolas et al., 2021). Hence, we explored the correlation of GARS with the immune microenvironment and immunotherapy response.

Immunotherapy is a novel treatment for LUAD. Identifying a novel method that can classify patients who might benefit from immunotherapy remains a tremendous challenge (Camidge et al., 2019; Yu et al., 2019; Yu et al., 2020; Wu et al., 2021). Recent studies have demonstrated numerous differences between the GA and the immune microenvironment; the GA can participate in the activation of the NLRP3 inflammasome, sensing nucleic acids, and influencing interferon (IFN)-inducible GTPases (Vey et al., 1994; Gonugunta et al., 2017; Chen and Chen, 2018), highlighting its potential in predicting immune-related anticancer therapy. In this study, we found that the immune response was significantly higher in the low GARS group, including lower immune cell infiltration (such as CD4^+^ T cells, CD8^+^ T cells, NK cells, monocytes, M0 macrophages, M2 macrophages, and B cells), immune pathway response, gene expression of immune checkpoints, immune score, stromal score, and ESTIMATE score. These results illustrate that lower immune activities were revealed in the high GARS group. At present, researchers investigating LUAD patients who might respond to immunotherapy mainly relied on IPS (Guo et al., 2021); however, few studies have been validated using a direct lung cancer cohort. To further explore whether GARS can predict the efficacy of immunotherapy, this study not only administered IPS score to forecast but also used GSE126044 and GSE13522 to direct confirmation. These results strongly verified the potential of GARS in predicting the efficacy of LUAD immunotherapy. In addition, the most significant GO and KEGG pathways between high and low GARS are the humoral immune response, external side plasma membrane, antigen binding, and complement and coagulation cascades. This indicates that the most important LUAD prognosis-related function of GA is its intimate connection with immune response. Taken together, these results suggest that patients in the low GARS group may respond better to immunotherapy.

The GARS constructed in this study may also instruct clinicians to select other anticancer drugs for the treatment of LUAD. In this study, a high GARS was significantly correlated with high drug sensitivity, including cisplatin, docetaxel, erlotinib, etoposide, gemcitabine, paclitaxel, rapamycin, sorafenib, and vinorelbine. Among them, cisplatin, docetaxel, etoposide, gemcitabine, paclitaxel, and vinorelbine are traditional chemotherapeutic drugs that have been proven to be beneficial in LUAD (I. Kim et al., 2019; Z. Li Y. et al., 2021; Perdrizet et al., 2021; Provencio et al., 2021; Yamaguchi et al., 2021). Erlotinib, the first approved target drug for NSCLC, is a reversible tyrosine kinase inhibitor that targets the epidermal growth factor receptor and can significantly improve the survival of LUAD patients (Faehling et al., 2018). In addition, rapamycin (also called sirolimus) and sorafenib also have the potential for treating LUAD patients (Moran et al., 2017; Lim et al., 2016). Hence, the GARS we constructed may also instruct clinicians to select appropriate drugs for the treatment of LUAD.

In addition, to better guide clinical application, a nomogram was established to predict the prognosis of LUAD patients. This table also indicates the importance of GARS in predicting the survival of LUAD patients.

This study had some limitations. Because the proportion of late-stage patients is lower, the prediction ability of this GARS formula for late-stage LUAD is ambiguous. Although the study can stratify patients with stage IA LUAD, stage IA patients usually only received surgical therapy. Therefore, GARS may not be able to precisely predict further treatment in stage IA LUAD patients based on this risk stratification.

## Conclusion

In conclusion, the present study is the first to administer the GA gene signature to construct a GARS that exhibited high diagnostic accuracy in predicting prognosis in patients with LUAD. Functionally, the GARS was related to the TP53 mutation, TTN mutation, activation of immune microenvironment, the effect of immunotherapy, and multiple anticancer drugs in LUAD patients. We also constructed an accessible nomogram for clinical applications. In addition, this study may also provide direction for scientific research on GA and tumor development.

## Data Availability

Publicly available datasets were analyzed in this study. This data can be found here: TCGA-LUAD from TCGA database (https://portal.gdc.cancer.gov/); GSE68465, GSE72094, GSE50081, GSE135222 and GSE126044 cohort from GEO database (https://www.ncbi.nlm.nih.gov/geo/).
