# Supplementary material for: Prognosis and Immunotherapy Response With a Novel Golgi Apparatus Signature-Based Formula in Lung Adenocarcinoma
Source: Front Cell Dev Biol. 2022 Jan 20;9:817085. doi: 10.3389/fcell.2021.817085 (PMC8811463; doi:10.3389/fcell.2021.817085)
Supplement: Supplementary file 1 [file Table1.PDF]

Supplementary Table 1

(A)GO enrichment analysis of Golgi Apparatus related DEGs

Catagroy	ID	Description	Counts	qvalue
BP	GO:0009101	glycoprotein biosynthetic process	65	5.70E-45
BP	GO:0009100	glycoprotein metabolic process	66	7.57E-41
BP	GO:0070085	glycosylation	48	8.64E-32
BP	GO:0006486	protein glycosylation	47	8.64E-32
BP	GO:0043413	macromolecule glycosylation	47	8.64E-32
BP	GO:0006023	aminoglycan biosynthetic process	30	3.01E-24
BP	GO:0006024	glycosaminoglycan biosynthetic process	28	1.53E-22
BP	GO:0016266	O-glycan processing	23	1.53E-22
BP	GO:0006493	protein O-linked glycosylation	27	1.58E-21
BP	GO:0006022	aminoglycan metabolic process	32	3.02E-21
BP	GO:0030203	glycosaminoglycan metabolic process	30	3.87E-20
BP	GO:1903510	mucopolysaccharide metabolic process	21	1.50E-13
BP	GO:0030166	proteoglycan biosynthetic process	15	5.89E-11
BP	GO:0006029	proteoglycan metabolic process	16	8.30E-10
BP	GO:0009311	oligosaccharide metabolic process	13	1.10E-09
BP	GO:0097503	sialylation	9	3.90E-09
BP	GO:0016051	carbohydrate biosynthetic process	21	3.32E-08
BP	GO:0044272	sulfur compound biosynthetic process	20	3.32E-08
BP	GO:0006672	ceramide metabolic process	15	3.62E-08

BP	GO:0006688	glycosphingolipid biosynthetic process	9	5.29E-08
BP	GO:0006027	glycosaminoglycan catabolic process	12	8.24E-08
BP	GO:0048193	Golgi vesicle transport	27	8.24E-08
BP	GO:0006888	endoplasmic reticulum to Golgi vesicle-mediated transport	20	9.15E-08
BP	GO:0018146	keratan sulfate biosynthetic process	9	9.39E-08
BP	GO:0001574	ganglioside biosynthetic process	7	9.39E-08
BP	GO:0006643	membrane lipid metabolic process	20	1.34E-07
BP	GO:0006026	aminoglycan catabolic process	12	2.03E-07
BP	GO:0050650	chondroitin sulfate proteoglycan biosynthetic process	9	2.26E-07
BP	GO:0007599	hemostasis	25	3.07E-07
BP	GO:0050817	coagulation	25	3.14E-07
BP	GO:0042339	keratan sulfate metabolic process	9	3.78E-07
BP	GO:0006665	sphingolipid metabolic process	17	4.33E-07
BP	GO:0009312	oligosaccharide biosynthetic process	7	7.59E-07
BP	GO:0030206	chondroitin sulfate biosynthetic process	8	8.90E-07
BP	GO:0007596	blood coagulation	24	8.90E-07
BP	GO:0046513	ceramide biosynthetic process	11	1.90E-06
BP	GO:0050818	regulation of coagulation	12	3.66E-06
BP	GO:0030148	sphingolipid biosynthetic process	13	3.99E-06
BP	GO:0050654	chondroitin sulfate proteoglycan metabolic process	9	4.66E-06
BP	GO:0001573	ganglioside metabolic process	7	5.09E-06
BP	GO:0006687	glycosphingolipid metabolic process	10	1.40E-05

BP	GO:0030193	regulation of blood coagulation	11	1.41E-05
BP	GO:0009247	glycolipid biosynthetic process	10	1.52E-05
BP	GO:1900046	regulation of hemostasis	11	1.52E-05
BP	GO:0036065	fucosylation	6	2.03E-05
BP	GO:0030204	chondroitin sulfate metabolic process	8	2.14E-05
BP	GO:0046467	membrane lipid biosynthetic process	14	2.15E-05
BP	GO:0061041	regulation of wound healing	14	2.93E-05
BP	GO:0006664	glycolipid metabolic process	12	3.35E-05
BP	GO:0030198	extracellular matrix organization	23	3.49E-05
BP	GO:1903509	liposaccharide metabolic process	12	3.50E-05
BP	GO:0043062	extracellular structure organization	23	3.50E-05
BP	GO:0003093	regulation of glomerular filtration	5	4.02E-05
BP	GO:0006790	sulfur compound metabolic process	22	4.22E-05
BP	GO:1903034	regulation of response to wounding	15	4.80E-05
BP	GO:0003073	regulation of systemic arterial blood pressure	11	4.80E-05
BP	GO:0031214	biomineral tissue development	14	6.01E-05
BP	GO:0110148	biomineralization	14	6.01E-05
BP	GO:0030208	dermatan sulfate biosynthetic process	5	6.01E-05
BP	GO:0001503	ossification	23	6.01E-05
BP	GO:0043434	response to peptide hormone	24	6.86E-05
BP	GO:0030205	dermatan sulfate metabolic process	5	9.29E-05
BP	GO:0016486	peptide hormone processing	7	0.000101

BP	GO:0098801	regulation of renal system process	7	0.000101
BP	GO:0140448	signaling receptor ligand precursor processing	7	0.000101
BP	GO:0003094	glomerular filtration	6	0.000136
BP	GO:0097205	renal filtration	6	0.000173
BP	GO:0042391	regulation of membrane potential	23	0.00018
BP	GO:0050651	dermatan sulfate proteoglycan biosynthetic process	5	0.000189
BP	GO:0030168	platelet activation	13	0.000198
BP	GO:0031349	positive regulation of defense response	21	0.000214
BP	GO:0045089	positive regulation of innate immune response	15	0.000247
BP	GO:0001977	renal system process involved in regulation of blood volume	5	0.00025
BP	GO:0006004	fucose metabolic process	5	0.00025
BP	GO:0050655	dermatan sulfate proteoglycan metabolic process	5	0.00025
BP	GO:0002758	innate immune response-activating signal transduction	11	0.000279
BP	GO:0002218	activation of innate immune response	12	0.000369
BP	GO:0002833	positive regulation of response to biotic stimulus	16	0.000391
BP	GO:0016485	protein processing	15	0.000391
BP	GO:0051896	regulation of protein kinase B signaling	16	0.000426
BP	GO:0001822	kidney development	17	0.000435
BP	GO:0006491	N-glycan processing	5	0.000435
BP	GO:0072001	renal system development	17	0.000644
BP	GO:0014068	positive regulation of phosphatidylinositol 3-kinase signaling	9	0.000887
BP	GO:0009306	protein secretion	22	0.000895
BP	GO:0035592	establishment of protein localization to extracellular region	22	0.000914

BP	GO:0018209	peptidyl-serine modification	18	0.00094<br>2
BP	GO:0070374	positive regulation of ERK1 and ERK2 cascade	14	0.00099<br>5
BP	GO:0002223	stimulatory C-type lectin receptor signaling pathway	10	0.00106<br>1
BP	GO:0071692	protein localization to extracellular region	22	0.00109<br>6
BP	GO:0003018	vascular process in circulatory system	13	0.00110<br>4
BP	GO:0001508	action potential	11	0.00110<br>4
BP	GO:0031667	response to nutrient levels	22	0.00115<br>5
BP	GO:0043491	protein kinase B signaling	16	0.00115<br>5
BP	GO:0048015	phosphatidylinositol-mediated signaling	13	0.00118<br>5
BP	GO:0061097	regulation of protein tyrosine kinase activity	9	0.00121
BP	GO:0002220	innate immune response activating cell surface receptor signaling pathway	10	0.00122<br>1
BP	GO:0030282	bone mineralization	10	0.00128<br>3
BP	GO:0001765	membrane raft assembly	4	0.00128<br>3
BP	GO:0030311	poly-N-acetyllactosamine biosynthetic process	4	0.00128<br>3
BP	GO:0071560	cellular response to transforming growth factor beta stimulus	15	0.00128<br>3
BP	GO:1901136	carbohydrate derivative catabolic process	13	0.00129<br>7
BP	GO:0048017	inositol lipid-mediated signaling	13	0.00135<br>4
BP	GO:0043627	response to estrogen	8	0.00150<br>1
BP	GO:0044273	sulfur compound catabolic process	7	0.00158
BP	GO:0071559	response to transforming growth factor beta	15	0.00159<br>9
BP	GO:0030309	poly-N-acetyllactosamine metabolic process	4	0.00170<br>9
BP	GO:0003071	renal system process involved in regulation of systemic arterial blood pressure	5	0.00184<br>8

BP	GO:0051480	regulation of cytosolic calcium ion concentration	18	0.001848
BP	GO:0051604	protein maturation	16	0.001848
BP	GO:0000271	polysaccharide biosynthetic process	8	0.001868
BP	GO:0044262	cellular carbohydrate metabolic process	16	0.001889
BP	GO:0001655	urogenital system development	17	0.002197
BP	GO:0030207	chondroitin sulfate catabolic process	4	0.002215
BP	GO:0061098	positive regulation of protein tyrosine kinase activity	7	0.002263
BP	GO:0009612	response to mechanical stimulus	13	0.00232
BP	GO:0090303	positive regulation of wound healing	7	0.002476
BP	GO:0070372	regulation of ERK1 and ERK2 cascade	16	0.002863
BP	GO:0030194	positive regulation of blood coagulation	5	0.002924
BP	GO:1900048	positive regulation of hemostasis	5	0.002924
BP	GO:0016050	vesicle organization	17	0.002924
BP	GO:0051047	positive regulation of secretion	17	0.002924
BP	GO:0008217	regulation of blood pressure	12	0.003036
BP	GO:0006939	smooth muscle contraction	9	0.003251
BP	GO:0034637	cellular carbohydrate biosynthetic process	8	0.003325
BP	GO:0050820	positive regulation of coagulation	5	0.00335
BP	GO:1903532	positive regulation of secretion by cell	16	0.003438
BP	GO:0072659	protein localization to plasma membrane	15	0.003438
BP	GO:0048207	vesicle targeting, rough ER to cis-Golgi	7	0.003691
BP	GO:0048208	COPII vesicle coating	7	0.003691
BP	GO:0050804	modulation of chemical synaptic transmission	20	0.003901

BP	GO:0099177	regulation of trans-synaptic signaling	20	0.003986
BP	GO:0044764	multi-organism cellular process	4	0.00433
BP	GO:0031623	receptor internalization	9	0.004527
BP	GO:0033273	response to vitamin	8	0.004571
BP	GO:0003254	regulation of membrane depolarization	6	0.00473
BP	GO:0070371	ERK1 and ERK2 cascade	16	0.004897
BP	GO:0035296	regulation of tube diameter	10	0.004897
BP	GO:0097746	regulation of blood vessel diameter	10	0.004897
BP	GO:0071295	cellular response to vitamin	5	0.004897
BP	GO:0006901	vesicle coating	7	0.004934
BP	GO:0035150	regulation of tube size	10	0.005061
BP	GO:0001649	osteoblast differentiation	13	0.005061
BP	GO:0051923	sulfation	4	0.005061
BP	GO:0050714	positive regulation of protein secretion	11	0.005061
BP	GO:0010959	regulation of metal ion transport	18	0.005061
BP	GO:0033692	cellular polysaccharide biosynthetic process	7	0.005172
BP	GO:0070838	divalent metal ion transport	20	0.005539
BP	GO:0070167	regulation of biomineral tissue development	8	0.005539
BP	GO:0110149	regulation of biomineralization	8	0.005539
BP	GO:0090114	COPII-coated vesicle budding	7	0.005963
BP	GO:0006044	N-acetylglucosamine metabolic process	4	0.005963
BP	GO:0010561	negative regulation of glycoprotein biosynthetic process	4	0.005963
BP	GO:0048199	vesicle targeting, to, from or within	7	0.00634

		Golgi		7
BP	GO:1903036	positive regulation of response to wounding	7	0.00634<br>7
BP	GO:0072511	divalent inorganic cation transport	20	0.00641<br>6
BP	GO:0015909	long-chain fatty acid transport	7	0.00680<br>8
BP	GO:1990778	protein localization to cell periphery	16	0.00703<br>8
BP	GO:0003014	renal system process	9	0.00712<br>1
BP	GO:0045088	regulation of innate immune response	15	0.00712<br>7
BP	GO:0042326	negative regulation of phosphorylation	20	0.00728
BP	GO:0045807	positive regulation of endocytosis	8	0.00728
BP	GO:0010543	regulation of platelet activation	5	0.00743<br>6
BP	GO:0050819	negative regulation of coagulation	6	0.00744<br>6
BP	GO:0014065	phosphatidylinositol 3-kinase signaling	10	0.00752<br>3
BP	GO:0042632	cholesterol homeostasis	8	0.0076
BP	GO:0014066	regulation of phosphatidylinositol 3-kinase signaling	9	0.00762<br>3
BP	GO:0055092	sterol homeostasis	8	0.00802<br>7
BP	GO:0001502	cartilage condensation	4	0.00806
BP	GO:0030277	maintenance of gastrointestinal epithelium	4	0.00806
BP	GO:0050708	regulation of protein secretion	16	0.00943<br>9
BP	GO:0021516	dorsal spinal cord development	4	0.00943<br>9
BP	GO:0051900	regulation of mitochondrial depolarization	4	0.00943<br>9
BP	GO:0055093	response to hyperoxia	4	0.00943<br>9
BP	GO:1903019	negative regulation of glycoprotein metabolic process	4	0.00943<br>9
BP	GO:0019751	polyol metabolic process	9	0.00959<br>5
BP	GO:0048194	Golgi vesicle budding	7	0.00967<br>5
BP	GO:0051222	positive regulation of protein transport	16	0.00970

				1
BP	GO:0006816	calcium ion transport	18	0.009846
BP	GO:0018242	protein O-linked glycosylation via serine	3	0.010033
BP	GO:0035860	glial cell-derived neurotrophic factor receptor signaling pathway	3	0.010033
BP	GO:0044857	plasma membrane raft organization	3	0.010033
BP	GO:0072593	reactive oxygen species metabolic process	14	0.01021
BP	GO:0002793	positive regulation of peptide secretion	11	0.010463
BP	GO:0019229	regulation of vasoconstriction	6	0.010463
BP	GO:0097755	positive regulation of blood vessel diameter	6	0.010463
BP	GO:0018230	peptidyl-L-cysteine S-palmitoylation	4	0.010463
BP	GO:0018231	peptidyl-S-diacylglycerol-L-cysteine biosynthetic process from peptidyl-cysteine	4	0.010463
BP	GO:0007229	integrin-mediated signaling pathway	8	0.010463
BP	GO:0031589	cell-substrate adhesion	16	0.010567
BP	GO:0071496	cellular response to external stimulus	15	0.011138
BP	GO:0006936	muscle contraction	16	0.011429
BP	GO:1901653	cellular response to peptide	17	0.011454
BP	GO:0001894	tissue homeostasis	13	0.011908
BP	GO:0051882	mitochondrial depolarization	4	0.011908
BP	GO:0098743	cell aggregation	4	0.011908
BP	GO:0006836	neurotransmitter transport	12	0.012015
BP	GO:0043112	receptor metabolic process	11	0.012198
BP	GO:0042985	negative regulation of amyloid precursor protein biosynthetic process	3	0.012391

BP	GO:0051673	membrane disruption in other organism	3	0.012391
BP	GO:0005976	polysaccharide metabolic process	8	0.012511
BP	GO:0010876	lipid localization	18	0.012672
BP	GO:0032963	collagen metabolic process	8	0.013068
BP	GO:0035821	modulation of process of other organism	8	0.013068
BP	GO:0007586	digestion	9	0.013137
BP	GO:0032355	response to estradiol	9	0.013137
BP	GO:0001763	morphogenesis of a branching structure	11	0.013137
BP	GO:0031099	regeneration	11	0.013137
BP	GO:1904951	positive regulation of establishment of protein localization	16	0.013235
BP	GO:0001933	negative regulation of protein phosphorylation	18	0.013513
BP	GO:0050806	positive regulation of synaptic transmission	10	0.013913
BP	GO:0001505	regulation of neurotransmitter levels	12	0.013913
BP	GO:0002831	regulation of response to biotic stimulus	17	0.013913
BP	GO:0048640	negative regulation of developmental growth	8	0.013913
BP	GO:0006940	regulation of smooth muscle contraction	6	0.013913
BP	GO:0031646	positive regulation of nervous system process	6	0.013913
BP	GO:0060348	bone development	11	0.014211
BP	GO:0031670	cellular response to nutrient	5	0.014283
BP	GO:0060070	canonical Wnt signaling pathway	15	0.014431
BP	GO:0031579	membrane raft organization	4	0.014454
BP	GO:1901071	glucosamine-containing compound metabolic process	4	0.014454

BP	GO:0042340	keratan sulfate catabolic process	3	0.014626
BP	GO:1904953	Wnt signaling pathway involved in midbrain dopaminergic neuron differentiation	3	0.014626
BP	GO:0014902	myotube differentiation	8	0.014933
BP	GO:0051899	membrane depolarization	7	0.015146
BP	GO:0014047	glutamate secretion	5	0.015146
BP	GO:0032365	intracellular lipid transport	5	0.015146
BP	GO:1904646	cellular response to amyloid-beta	5	0.015146
BP	GO:0072503	cellular divalent inorganic cation homeostasis	19	0.015952
BP	GO:0002021	response to dietary excess	4	0.016081
BP	GO:0002791	regulation of peptide secretion	16	0.016259
BP	GO:0006874	cellular calcium ion homeostasis	18	0.016517
BP	GO:0030100	regulation of endocytosis	11	0.016679
BP	GO:1903035	negative regulation of response to wounding	7	0.016679
BP	GO:0006979	response to oxidative stress	18	0.017122
BP	GO:0060993	kidney morphogenesis	7	0.017457
BP	GO:1901264	carbohydrate derivative transport	7	0.017457
BP	GO:1905954	positive regulation of lipid localization	7	0.017457
BP	GO:0010669	epithelial structure maintenance	4	0.017586
BP	GO:0072376	protein activation cascade	4	0.017586
BP	GO:0072378	blood coagulation, fibrin clot formation	4	0.017586
BP	GO:1902932	positive regulation of alcohol biosynthetic process	4	0.017586
BP	GO:0022604	regulation of cell morphogenesis	19	0.01769

				5
BP	GO:0006903	vesicle targeting	7	0.01809<br>9
BP	GO:0032370	positive regulation of lipid transport	6	0.01809<br>9
BP	GO:0030177	positive regulation of Wnt signaling pathway	10	0.01906<br>2
BP	GO:0042982	amyloid precursor protein metabolic process	6	0.01932<br>5
BP	GO:0032868	response to insulin	13	0.01950<br>2
BP	GO:0036296	response to increased oxygen levels	4	0.01955<br>5
BP	GO:0018198	peptidyl-cysteine modification	5	0.02035<br>3
BP	GO:0055074	calcium ion homeostasis	18	0.02035<br>3
BP	GO:0006869	lipid transport	16	0.02048<br>3
BP	GO:0021527	spinal cord association neuron differentiation	3	0.02059
BP	GO:0035437	maintenance of protein localization in endoplasmic reticulum	3	0.02059
BP	GO:0060732	positive regulation of inositol phosphate biosynthetic process	3	0.02059
BP	GO:0048754	branching morphogenesis of an epithelial tube	9	0.02059
BP	GO:0001657	ureteric bud development	7	0.02059
BP	GO:0043409	negative regulation of MAPK cascade	10	0.02101<br>3
BP	GO:0061138	morphogenesis of a branching epithelium	10	0.02101<br>3
BP	GO:0010517	regulation of phospholipase activity	6	0.02101<br>3
BP	GO:0051924	regulation of calcium ion transport	12	0.02101<br>3
BP	GO:0070482	response to oxygen levels	16	0.02101<br>3
BP	GO:0003044	regulation of systemic arterial blood pressure mediated by a chemical signal	5	0.02101<br>3
BP	GO:0030195	negative regulation of blood coagulation	5	0.02101<br>3
BP	GO:0050830	defense response to Gram-positive	7	0.02101

		bacterium		3
BP	GO:0072163	mesonephric epithelium development	7	0.021013
BP	GO:0072164	mesonephric tubule development	7	0.021013
BP	GO:0042176	regulation of protein catabolic process	16	0.02134
BP	GO:0031669	cellular response to nutrient levels	11	0.021668
BP	GO:0042445	hormone metabolic process	11	0.021668
BP	GO:0050435	amyloid-beta metabolic process	5	0.022409
BP	GO:1900047	negative regulation of hemostasis	5	0.022409
BP	GO:0043687	post-translational protein modification	15	0.022667
BP	GO:0002576	platelet degranulation	8	0.022717
BP	GO:0015908	fatty acid transport	7	0.022717
BP	GO:0044264	cellular polysaccharide metabolic process	7	0.022717
BP	GO:0060191	regulation of lipase activity	7	0.022717
BP	GO:0006898	receptor-mediated endocytosis	14	0.023147
BP	GO:0034638	phosphatidylcholine catabolic process	3	0.023168
BP	GO:1900225	regulation of NLRP3 inflammasome complex assembly	3	0.023168
BP	GO:0022600	digestive system process	7	0.02369
BP	GO:1903524	positive regulation of blood circulation	6	0.024022
BP	GO:0032147	activation of protein kinase activity	14	0.024649
BP	GO:0001823	mesonephros development	7	0.024649
BP	GO:0030201	heparan sulfate proteoglycan metabolic process	4	0.024649
BP	GO:0050482	arachidonic acid secretion	4	0.024649
BP	GO:1903963	arachidonate transport	4	0.024649
BP	GO:0051051	negative regulation of transport	18	0.02496

				7
BP	GO:0045599	negative regulation of fat cell differentiation	5	0.024967
BP	GO:0030500	regulation of bone mineralization	6	0.024967
BP	GO:0045862	positive regulation of proteolysis	15	0.025452
BP	GO:0055088	lipid homeostasis	9	0.025452
BP	GO:1905475	regulation of protein localization to membrane	10	0.025544
BP	GO:0036293	response to decreased oxygen levels	15	0.025927
BP	GO:0042310	vasoconstriction	6	0.026116
BP	GO:0061045	negative regulation of wound healing	6	0.026116
BP	GO:0009620	response to fungus	5	0.02627
BP	GO:0010524	positive regulation of calcium ion transport into cytosol	5	0.02627
BP	GO:0010919	regulation of inositol phosphate biosynthetic process	3	0.02627
BP	GO:0010934	macrophage cytokine production	3	0.02627
BP	GO:0034765	regulation of ion transmembrane transport	18	0.027211
BP	GO:0018108	peptidyl-tyrosine phosphorylation	15	0.027211
BP	GO:0071260	cellular response to mechanical stimulus	6	0.027211
BP	GO:0007218	neuropeptide signaling pathway	7	0.027236
BP	GO:0009743	response to carbohydrate	11	0.028818
BP	GO:0018212	peptidyl-tyrosine modification	15	0.028818
BP	GO:0043276	anoikis	4	0.028818
BP	GO:0045907	positive regulation of vasoconstriction	4	0.028818
BP	GO:0097305	response to alcohol	11	0.029398
BP	GO:0008593	regulation of Notch signaling pathway	7	0.029398
BP	GO:0019320	hexose catabolic process	5	0.02939

				8
BP	GO:0060688	regulation of morphogenesis of a branching structure	5	0.029398
BP	GO:0086002	cardiac muscle cell action potential involved in contraction	5	0.029398
BP	GO:1904645	response to amyloid-beta	5	0.029398
BP	GO:0050829	defense response to Gram-negative bacterium	6	0.029581
BP	GO:0000302	response to reactive oxygen species	11	0.029581
BP	GO:0044546	NLRP3 inflammasome complex assembly	3	0.029581
BP	GO:0045986	negative regulation of smooth muscle contraction	3	0.029581
BP	GO:1904948	midbrain dopaminergic neuron differentiation	3	0.029581
BP	GO:0006638	neutral lipid metabolic process	8	0.029581
BP	GO:0006639	acylglycerol metabolic process	8	0.029581
BP	GO:0006900	vesicle budding from membrane	7	0.031648
BP	GO:0007584	response to nutrient	9	0.031715
BP	GO:0010769	regulation of cell morphogenesis involved in differentiation	13	0.032815
BP	GO:0018105	peptidyl-serine phosphorylation	13	0.032815
BP	GO:0018345	protein palmitoylation	4	0.033441
BP	GO:0031128	developmental induction	4	0.033441
BP	GO:0071402	cellular response to lipoprotein particle stimulus	4	0.033441
BP	GO:0006937	regulation of muscle contraction	9	0.033467
BP	GO:0006677	glycosylceramide metabolic process	3	0.033467
BP	GO:0042983	amyloid precursor protein biosynthetic process	3	0.033467
BP	GO:0042984	regulation of amyloid precursor protein biosynthetic process	3	0.033467
BP	GO:1990000	amyloid fibril formation	3	0.03346

				7
BP	GO:0045913	positive regulation of carbohydrate metabolic process	6	0.033467
BP	GO:0050886	endocrine process	6	0.033467
BP	GO:0022898	regulation of transmembrane transporter activity	12	0.033676
BP	GO:0034284	response to monosaccharide	10	0.034
BP	GO:0010469	regulation of signaling receptor activity	9	0.034
BP	GO:0019731	antibacterial humoral response	5	0.0341
BP	GO:0032387	negative regulation of intracellular transport	5	0.0341
BP	GO:0052547	regulation of peptidase activity	17	0.034269
BP	GO:0032092	positive regulation of protein binding	6	0.034763
BP	GO:0003012	muscle system process	17	0.034807
BP	GO:0098926	postsynaptic signal transduction	4	0.035264
BP	GO:0030900	forebrain development	15	0.036084
BP	GO:0097756	negative regulation of blood vessel diameter	6	0.036406
BP	GO:0038127	ERBB signaling pathway	8	0.036985
BP	GO:0042593	glucose homeostasis	11	0.037076
BP	GO:0002076	osteoblast development	3	0.037329
BP	GO:0010544	negative regulation of platelet activation	3	0.037329
BP	GO:0035988	chondrocyte proliferation	3	0.037329
BP	GO:0043393	regulation of protein binding	10	0.037329
BP	GO:0033500	carbohydrate homeostasis	11	0.037364
BP	GO:0072330	monocarboxylic acid biosynthetic process	11	0.037364
BP	GO:0051897	positive regulation of protein kinase B signaling	9	0.037364
BP	GO:0002385	mucosal immune response	4	0.037364

BP	GO:0060306	regulation of membrane repolarization	4	0.037364
BP	GO:0044344	cellular response to fibroblast growth factor stimulus	8	0.037364
BP	GO:0007568	aging	13	0.037728
BP	GO:0051650	establishment of vesicle localization	10	0.037728
BP	GO:0050678	regulation of epithelial cell proliferation	15	0.037838
BP	GO:0046486	glycerolipid metabolic process	16	0.037838
BP	GO:0044409	entry into host	8	0.038392
BP	GO:0030073	insulin secretion	10	0.038561
BP	GO:0009798	axis specification	6	0.038642
BP	GO:0043270	positive regulation of ion transport	12	0.038778
BP	GO:0010518	positive regulation of phospholipase activity	5	0.039026
BP	GO:0055081	anion homeostasis	5	0.039026
BP	GO:0001666	response to hypoxia	14	0.039085
BP	GO:0008277	regulation of G protein-coupled receptor signaling pathway	8	0.039122
BP	GO:0031644	regulation of nervous system process	8	0.039122
CC	GO:0005796	Golgi lumen	49	8.78E-56
CC	GO:0098791	Golgi apparatus subcompartment	54	2.73E-31
CC	GO:0031984	organelle subcompartment	54	4.96E-30
CC	GO:0005802	trans-Golgi network	31	9.26E-16
CC	GO:0005795	Golgi stack	24	1.98E-14
CC	GO:0005788	endoplasmic reticulum lumen	30	2.70E-12
CC	GO:0030133	transport vesicle	34	5.10E-12

CC	GO:0005798	Golgi-associated vesicle	23	7.28E-12
CC	GO:0031985	Golgi cisterna	19	9.44E-12
CC	GO:0032580	Golgi cisterna membrane	17	2.07E-11
CC	GO:0062023	collagen-containing extracellular matrix	31	1.26E-09
CC	GO:0030135	coated vesicle	24	2.44E-08
CC	GO:0030658	transport vesicle membrane	20	2.96E-08
CC	GO:0030660	Golgi-associated vesicle membrane	15	3.24E-08
CC	GO:0031228	intrinsic component of Golgi membrane	12	3.68E-08
CC	GO:0030666	endocytic vesicle membrane	16	8.31E-07
CC	GO:0030173	integral component of Golgi membrane	10	2.06E-06
CC	GO:0030662	coated vesicle membrane	16	3.40E-06
CC	GO:0030139	endocytic vesicle	21	3.96E-06
CC	GO:0005769	early endosome	23	5.49E-06
CC	GO:0005801	cis-Golgi network	10	6.36E-06
CC	GO:0045121	membrane raft	21	7.65E-06
CC	GO:0098857	membrane microdomain	21	7.65E-06
CC	GO:0005793	endoplasmic reticulum-Golgi intermediate compartment	13	7.65E-06
CC	GO:0098589	membrane region	21	1.33E-05
CC	GO:0043202	lysosomal lumen	11	1.33E-05
CC	GO:0005901	caveola	10	2.21E-05
CC	GO:0031300	intrinsic component of organelle membrane	22	3.57E-05
CC	GO:0033116	endoplasmic reticulum-Golgi intermediate compartment membrane	9	7.62E-05

CC	GO:0030134	COPII-coated ER to Golgi transport vesicle	10	7.62E-05
CC	GO:0030136	clathrin-coated vesicle	14	0.000107
CC	GO:0005775	vacuolar lumen	13	0.000138
CC	GO:0044853	plasma membrane raft	10	0.00032
CC	GO:0030665	clathrin-coated vesicle membrane	10	0.00045
CC	GO:0043195	terminal bouton	7	0.000501
CC	GO:0044306	neuron projection terminus	11	0.000645
CC	GO:0005791	rough endoplasmic reticulum	8	0.00102
CC	GO:0030140	trans-Golgi network transport vesicle	5	0.001826
CC	GO:0031225	anchored component of membrane	11	0.002006
CC	GO:0032588	trans-Golgi network membrane	8	0.002063
CC	GO:0008021	synaptic vesicle	12	0.002432
CC	GO:0031301	integral component of organelle membrane	17	0.002542
CC	GO:0043679	axon terminus	9	0.003634
CC	GO:0030672	synaptic vesicle membrane	8	0.003634
CC	GO:0099501	exocytic vesicle membrane	8	0.003634
CC	GO:0046658	anchored component of plasma membrane	6	0.004377
CC	GO:0070382	exocytic vesicle	12	0.004491
CC	GO:0031091	platelet alpha granule	7	0.007374
CC	GO:0031093	platelet alpha granule lumen	6	0.007374
CC	GO:0030130	clathrin coat of trans-Golgi network vesicle	3	0.008095
CC	GO:0036020	endolysosome membrane	3	0.009967
CC	GO:0012510	trans-Golgi network transport vesicle membrane	3	0.012057
CC	GO:0055037	recycling endosome	10	0.01265

				3
CC	GO:0005767	secondary lysosome	3	0.0141
CC	GO:0030120	vesicle coat	5	0.016829
CC	GO:0030663	COPI-coated vesicle membrane	3	0.019291
CC	GO:0045334	clathrin-coated endocytic vesicle	5	0.020372
CC	GO:0043197	dendritic spine	9	0.021845
CC	GO:0044309	neuron spine	9	0.023116
CC	GO:0012507	ER to Golgi transport vesicle membrane	5	0.023947
CC	GO:0036019	endolysosome	3	0.024093
CC	GO:0030669	clathrin-coated endocytic vesicle membrane	4	0.025543
CC	GO:0016324	apical plasma membrane	14	0.026941
CC	GO:0060205	cytoplasmic vesicle lumen	13	0.028094
CC	GO:0031983	vesicle lumen	13	0.029059
CC	GO:0032590	dendrite membrane	4	0.031067
MF	GO:0016757	transferase activity, transferring glycosyl groups	43	6.29E-25
MF	GO:0016758	transferase activity, transferring hexosyl groups	34	9.23E-21
MF	GO:0008194	UDP-glycosyltransferase activity	28	9.61E-19
MF	GO:0008376	acetylgalactosaminyltransferase activity	16	1.24E-14
MF	GO:0008373	sialyltransferase activity	9	4.99E-09
MF	GO:0008375	acetylglucosaminyltransferase activity	11	1.35E-07
MF	GO:0008146	sulfotransferase activity	11	1.44E-07
MF	GO:0140103	catalytic activity, acting on a glycoprotein	8	2.78E-07
MF	GO:0004653	polypeptide N-acetylgalactosaminyltransferase	7	1.49E-06

		activity		
MF	GO:0016782	transferase activity, transferring sulfur-containing groups	11	2.41E-06
MF	GO:0008417	fucosyltransferase activity	6	2.41E-06
MF	GO:0005539	glycosaminoglycan binding	19	2.41E-06
MF	GO:0033218	amide binding	24	6.17E-06
MF	GO:0042277	peptide binding	21	9.46E-06
MF	GO:0030546	signaling receptor activator activity	27	1.42E-05
MF	GO:0048018	receptor ligand activity	26	3.67E-05
MF	GO:0001540	amyloid-beta binding	10	3.99E-05
MF	GO:0030246	carbohydrate binding	18	6.24E-05
MF	GO:0034483	heparan sulfate sulfotransferase activity	5	0.000135
MF	GO:0008378	galactosyltransferase activity	6	0.000191
MF	GO:0008532	N-acetyllactosaminide beta-1,3-N-acetylglucosaminyltransferase activity	4	0.000777
MF	GO:0005540	hyaluronic acid binding	5	0.000877
MF	GO:0030021	extracellular matrix structural constituent conferring compression resistance	5	0.000877
MF	GO:0035250	UDP-galactosyltransferase activity	5	0.001058
MF	GO:0005109	frizzled binding	6	0.001203
MF	GO:0005201	extracellular matrix structural constituent	12	0.001296
MF	GO:0030553	cGMP binding	4	0.002358
MF	GO:0031406	carboxylic acid binding	12	0.009873
MF	GO:0043177	organic acid binding	12	0.015004
MF	GO:0019838	growth factor binding	9	0.01500

				4
MF	GO:0008201	heparin binding	10	0.018148
MF	GO:0099106	ion channel regulator activity	8	0.023284
MF	GO:0019706	protein-cysteine S-palmitoyltransferase activity	4	0.023284
MF	GO:0019707	protein-cysteine S-acyltransferase activity	4	0.023284
MF	GO:0015562	efflux transmembrane transporter activity	3	0.026996
MF	GO:0005184	neuropeptide hormone activity	4	0.0286
MF	GO:0015020	glucuronosyltransferase activity	4	0.034547
MF	GO:0016417	S-acyltransferase activity	4	0.034547
MF	GO:0008083	growth factor activity	9	0.03859
MF	GO:1901681	sulfur compound binding	12	0.041336
MF	GO:0030169	low-density lipoprotein particle binding	3	0.041336

(B) KEGG enrichment analysis of Golgi Apparatus related DEGs

ID	Description	Count	qvalue
hsa00512	Mucin type O-Glycan biosynthesis	12	9.41E-10
hsa00601	Glycosphingolipid biosynthesis - lacto and neolacto series	10	4.51E-08
hsa00533	Glycosaminoglycan biosynthesis - keratan sulfate	6	6.94E-05
hsa00604	Glycosphingolipid biosynthesis - ganglio series	5	0.001143
hsa00534	Glycosaminoglycan biosynthesis - heparan sulfate / heparin	6	0.001487
hsa00532	Glycosaminoglycan biosynthesis - chondroitin sulfate / dermatan sulfate	5	0.005711

Supplementary Table 2

(A) GO enrichment analysis of high GARS and low GARS group patients

Category	ID	Description	Count	qvalue
BP	GO:0006959	humoral immune response	68	1.07E-22
BP	GO:0002455	humoral immune response mediated by circulating immunoglobulin	42	1.88E-20
BP	GO:0006958	complement activation, classical pathway	40	2.25E-20
BP	GO:0006956	complement activation	44	4.66E-20
BP	GO:0016064	immunoglobulin mediated immune response	45	1.61E-16
BP	GO:0019724	B cell mediated immunity	45	2.32E-16
BP	GO:0002449	lymphocyte mediated immunity	55	6.52E-15
BP	GO:0002460	adaptive immune response based on somatic recombination of immune receptors built from immunoglobulin superfamily domains	55	9.38E-15
BP	GO:0050853	B cell receptor signaling pathway	30	4.06E-12
BP	GO:0002920	regulation of humoral immune response	29	2.13E-11
BP	GO:0030449	regulation of complement activation	24	6.75E-09
BP	GO:0006910	phagocytosis, recognition	21	7.70E-09
BP	GO:0002377	immunoglobulin production	32	1.81E-08
BP	GO:0003341	cilium movement	27	2.82E-08
BP	GO:0002440	production of molecular mediator of immune response	39	3.30E-08
BP	GO:0042742	defense response to bacterium	42	3.38E-08
BP	GO:0002429	immune response-activating cell surface receptor signaling pathway	51	3.64E-08
BP	GO:0002757	immune response-activating signal transduction	51	3.64E-08
BP	GO:0001578	microtubule bundle formation	21	7.75E-08
BP	GO:0099024	plasma membrane invagination	24	1.24E-07
BP	GO:0002697	regulation of immune effector process	49	1.29E-07
BP	GO:0006911	phagocytosis, engulfment	23	1.47E-07
BP	GO:0019730	antimicrobial humoral response	24	1.47E-07
BP	GO:0010324	membrane invagination	24	3.47E-07
BP	GO:0140014	mitotic nuclear division	35	5.34E-07
BP	GO:0000070	mitotic sister chromatid segregation	25	7.78E-07
BP	GO:0006909	phagocytosis	41	1.12E-06
BP	GO:0007018	microtubule-based movement	40	1.16E-06
BP	GO:0042113	B cell activation	37	1.48E-06
BP	GO:0035082	axoneme assembly	16	1.48E-06
BP	GO:0050864	regulation of B cell activation	27	2.02E-06
BP	GO:0030071	regulation of mitotic metaphase/anaphase	14	3.39E-06

		transition		
BP	GO:0007091	metaphase/anaphase transition of mitotic cell cycle	14	5.18E-06
BP	GO:1902099	regulation of metaphase/anaphase transition of cell cycle	14	5.18E-06
BP	GO:0019731	antibacterial humoral response	14	6.35E-06
BP	GO:1902850	microtubule cytoskeleton organization involved in mitosis	22	6.35E-06
BP	GO:0044784	metaphase/anaphase transition of cell cycle	14	7.56E-06
BP	GO:0010965	regulation of mitotic sister chromatid separation	14	9.11E-06
BP	GO:0050851	antigen receptor-mediated signaling pathway	35	9.11E-06
BP	GO:0007088	regulation of mitotic nuclear division	23	1.29E-05
BP	GO:0050871	positive regulation of B cell activation	22	1.33E-05
BP	GO:0051306	mitotic sister chromatid separation	14	1.59E-05
BP	GO:0007052	mitotic spindle organization	19	1.84E-05
BP	GO:0051983	regulation of chromosome segregation	18	1.91E-05
BP	GO:1905818	regulation of chromosome separation	14	2.23E-05
BP	GO:0000819	sister chromatid segregation	25	2.35E-05
BP	GO:0008037	cell recognition	27	2.94E-05
BP	GO:0070286	axonemal dynein complex assembly	10	3.21E-05
BP	GO:0070268	cornification	18	3.99E-05
BP	GO:0007059	chromosome segregation	34	4.13E-05
BP	GO:0033047	regulation of mitotic sister chromatid segregation	14	6.09E-05
BP	GO:0033045	regulation of sister chromatid segregation	15	7.61E-05
BP	GO:0051783	regulation of nuclear division	23	0.000128
BP	GO:0048608	reproductive structure development	39	0.000222
BP	GO:0061458	reproductive system development	39	0.000271
BP	GO:0007051	spindle organization	22	0.000271
BP	GO:0051304	chromosome separation	15	0.000302
BP	GO:0007094	mitotic spindle assembly checkpoint	9	0.000343
BP	GO:0031577	spindle checkpoint	9	0.000343
BP	GO:0071173	spindle assembly checkpoint	9	0.000343
BP	GO:0071174	mitotic spindle checkpoint	9	0.000343
BP	GO:0033046	negative regulation of sister chromatid segregation	10	0.000449
BP	GO:0061844	antimicrobial humoral immune response mediated by antimicrobial peptide	13	0.000469
BP	GO:0000280	nuclear division	37	0.00051
BP	GO:0051985	negative regulation of chromosome segregation	10	0.000531
BP	GO:0045841	negative regulation of mitotic metaphase/anaphase transition	9	0.000531
BP	GO:1902100	negative regulation of metaphase/anaphase transition of cell cycle	9	0.000667
BP	GO:0050867	positive regulation of cell activation	36	0.000807

BP	GO:0048285	organelle fission	39	0.00094
BP	GO:2000816	negative regulation of mitotic sister chromatid separation	9	0.001013
BP	GO:1905819	negative regulation of chromosome separation	9	0.001243
BP	GO:0006690	icosanoid metabolic process	16	0.001507
BP	GO:0033048	negative regulation of mitotic sister chromatid segregation	9	0.001837
BP	GO:0090307	mitotic spindle assembly	11	0.002551
BP	GO:0006691	leukotriene metabolic process	8	0.003019
BP	GO:0031581	hemidesmosome assembly	5	0.003404
BP	GO:0036158	outer dynein arm assembly	6	0.003663
BP	GO:0002696	positive regulation of leukocyte activation	33	0.004338
BP	GO:0031638	zymogen activation	10	0.004492
BP	GO:0098813	nuclear chromosome segregation	25	0.004553
BP	GO:0051251	positive regulation of lymphocyte activation	30	0.004827
BP	GO:0019835	cytolysis	7	0.005637
BP	GO:0006898	receptor-mediated endocytosis	28	0.006128
BP	GO:0038095	Fc-epsilon receptor signaling pathway	18	0.00676
BP	GO:0007548	sex differentiation	25	0.006795
BP	GO:0045861	negative regulation of proteolysis	30	0.008169
BP	GO:0008544	epidermis development	36	0.008364
BP	GO:0045839	negative regulation of mitotic nuclear division	9	0.008834
BP	GO:0002431	Fc receptor mediated stimulatory signaling pathway	16	0.009762
BP	GO:0097529	myeloid leukocyte migration	21	0.009762
BP	GO:0036159	inner dynein arm assembly	5	0.009762
BP	GO:0031639	plasminogen activation	6	0.009963
BP	GO:0021988	olfactory lobe development	7	0.011853
BP	GO:0008608	attachment of spindle microtubules to kinetochore	7	0.014269
BP	GO:0007093	mitotic cell cycle checkpoint	17	0.014541
BP	GO:0045840	positive regulation of mitotic nuclear division	9	0.016605
BP	GO:0002433	immune response-regulating cell surface receptor signaling pathway involved in phagocytosis	15	0.017831
BP	GO:0038096	Fc-gamma receptor signaling pathway involved in phagocytosis	15	0.017831
BP	GO:0030647	aminoglycoside antibiotic metabolic process	4	0.017989
BP	GO:0051231	spindle elongation	4	0.017989
BP	GO:0070327	thyroid hormone transport	4	0.017989
BP	GO:0051302	regulation of cell division	17	0.019165
BP	GO:0007586	digestion	15	0.019772
BP	GO:0046456	icosanoid biosynthetic process	9	0.019956
BP	GO:0038094	Fc-gamma receptor signaling pathway	15	0.020912

BP	GO:0010466	negative regulation of peptidase activity	23	0.021589
BP	GO:0051784	negative regulation of nuclear division	9	0.022032
BP	GO:0052548	regulation of endopeptidase activity	32	0.022691
BP	GO:0008406	gonad development	20	0.022691
BP	GO:1901568	fatty acid derivative metabolic process	17	0.023195
BP	GO:0010951	negative regulation of endopeptidase activity	22	0.023923
BP	GO:0051225	spindle assembly	13	0.023923
BP	GO:0048875	chemical homeostasis within a tissue	4	0.024477
BP	GO:0002922	positive regulation of humoral immune response	5	0.026451
BP	GO:0033559	unsaturated fatty acid metabolic process	13	0.029778
BP	GO:0045137	development of primary sexual characteristics	20	0.02978
BP	GO:0031960	response to corticosteroid	16	0.029826
BP	GO:0050855	regulation of B cell receptor signaling pathway	6	0.029858
BP	GO:1902644	tertiary alcohol metabolic process	5	0.032695
BP	GO:0010755	regulation of plasminogen activation	4	0.032947
BP	GO:0035404	histone-serine phosphorylation	4	0.032947
BP	GO:0051255	spindle midzone assembly	4	0.032947
BP	GO:0052547	regulation of peptidase activity	33	0.032996
BP	GO:0120254	olefinic compound metabolic process	13	0.03497
BP	GO:0001539	cilium or flagellum-dependent cell motility	13	0.036866
BP	GO:0060285	cilium-dependent cell motility	13	0.036866
BP	GO:1990266	neutrophil migration	13	0.036866
BP	GO:0001909	leukocyte mediated cytotoxicity	12	0.038977
BP	GO:0030216	keratinocyte differentiation	24	0.039102
BP	GO:0007143	female meiotic nuclear division	6	0.039277
BP	GO:0001906	cell killing	16	0.044041
BP	GO:0001890	placenta development	15	0.044041
BP	GO:0051383	kinetochore organization	5	0.046535
BP	GO:0060294	cilium movement involved in cell motility	12	0.047299
CC	GO:0019814	immunoglobulin complex	53	1.90E-31
CC	GO:0009897	external side of plasma membrane	58	1.31E-14
CC	GO:0042571	immunoglobulin complex, circulating	22	1.35E-11
CC	GO:0042599	lamellar body	11	3.35E-10
CC	GO:0005930	axoneme	21	1.01E-06
CC	GO:0097014	ciliary plasm	21	1.14E-06
CC	GO:0031514	motile cilium	26	5.26E-06
CC	GO:0000779	condensed chromosome, centromeric region	19	1.58E-05
CC	GO:0062023	collagen-containing extracellular matrix	40	2.06E-05
CC	GO:0032838	plasma membrane bounded cell projection cytoplasm	24	0.00014
CC	GO:0000777	condensed chromosome kinetochore	16	0.00014
CC	GO:0031225	anchored component of membrane	21	0.00014
CC	GO:0000776	kinetochore	18	0.000232
CC	GO:0000940	condensed chromosome outer kinetochore	6	0.000232

CC	GO:0005874	microtubule	37	0.000249
CC	GO:0000775	chromosome, centromeric region	22	0.000272
CC	GO:0005875	microtubule associated complex	19	0.000411
CC	GO:0000780	condensed nuclear chromosome, centromeric region	8	0.000538
CC	GO:0000793	condensed chromosome	23	0.000538
CC	GO:0099568	cytoplasmic region	25	0.000556
CC	GO:0036157	outer dynein arm	5	0.000649
CC	GO:0016324	apical plasma membrane	31	0.000852
CC	GO:0005876	spindle microtubule	11	0.000852
CC	GO:0051233	spindle midzone	8	0.001037
CC	GO:0005819	spindle	31	0.001039
CC	GO:0005858	axonemal dynein complex	6	0.001314
CC	GO:0045177	apical part of cell	34	0.001838
CC	GO:0005771	multivesicular body	10	0.002019
CC	GO:0030286	dynein complex	9	0.002767
CC	GO:0000778	condensed nuclear chromosome kinetochore	5	0.004982
CC	GO:0000794	condensed nuclear chromosome	12	0.009442
CC	GO:0000922	spindle pole	16	0.010537
CC	GO:0097729	9+2 motile cilium	13	0.010904
CC	GO:0005922	connexin complex	5	0.012666
CC	GO:1904724	tertiary granule lumen	8	0.014709
CC	GO:0005921	gap junction	6	0.014769
CC	GO:1990023	mitotic spindle midzone	4	0.017886
CC	GO:0045334	clathrin-coated endocytic vesicle	8	0.021468
CC	GO:0031904	endosome lumen	6	0.021597
CC	GO:0005581	collagen trimer	10	0.021597
CC	GO:0072686	mitotic spindle	13	0.022366
MF	GO:0003823	antigen binding	40	1.14E-17
MF	GO:0034987	immunoglobulin receptor binding	22	1.01E-10
MF	GO:0005539	glycosaminoglycan binding	26	0.00039
MF	GO:0008201	heparin binding	21	0.000565
MF	GO:0004867	serine-type endopeptidase inhibitor activity	15	0.000897
MF	GO:0003777	microtubule motor activity	13	0.001024
MF	GO:0030414	peptidase inhibitor activity	21	0.001719
MF	GO:0071723	lipopeptide binding	5	0.001719
MF	GO:0033293	monocarboxylic acid binding	12	0.001721
MF	GO:0019865	immunoglobulin binding	7	0.001897
MF	GO:0050786	RAGE receptor binding	5	0.002163
MF	GO:0004866	endopeptidase inhibitor activity	20	0.002163
MF	GO:1901681	sulfur compound binding	25	0.002312
MF	GO:0048018	receptor ligand activity	38	0.002416
MF	GO:0008017	microtubule binding	25	0.002416
MF	GO:0004032	alditol:NADP+ 1-oxidoreductase activity	5	0.002529

MF	GO:0030546	signaling receptor activator activity	38	0.002632
MF	GO:0061135	endopeptidase regulator activity	20	0.002848
MF	GO:0061134	peptidase regulator activity	22	0.00404
MF	GO:0015631	tubulin binding	30	0.004386
MF	GO:1990939	ATP-dependent microtubule motor activity	7	0.009959
MF	GO:0005201	extracellular matrix structural constituent	17	0.011916
MF	GO:0032052	bile acid binding	4	0.012386
MF	GO:0008083	growth factor activity	16	0.019279
MF	GO:0003774	motor activity	14	0.019279
MF	GO:0005504	fatty acid binding	7	0.019279
MF	GO:0031406	carboxylic acid binding	19	0.019279
MF	GO:0005243	gap junction channel activity	5	0.026676
MF	GO:0008106	alcohol dehydrogenase (NADP+) activity	5	0.026676
MF	GO:0031994	insulin-like growth factor I binding	4	0.029408
MF	GO:0043177	organic acid binding	19	0.032276
MF	GO:0004089	carbonate dehydratase activity	4	0.049422

(B) KEGG enrichment analysis of high GARS group and low GARS group patients

ID	Description	Count	qvalue
hsa04610	Complement and coagulation cascades	15	0.000131
hsa04640	Hematopoietic cell lineage	15	0.000488
hsa05146	Amoebiasis	14	0.002198
