# Supplementary figures and images for: Prognosis and Immunotherapy Response With a Novel Golgi Apparatus Signature-Based Formula in Lung Adenocarcinoma
Source: Front Cell Dev Biol. 2022 Jan 20;9:817085. doi: 10.3389/fcell.2021.817085 (PMC8811463; doi:10.3389/fcell.2021.817085)

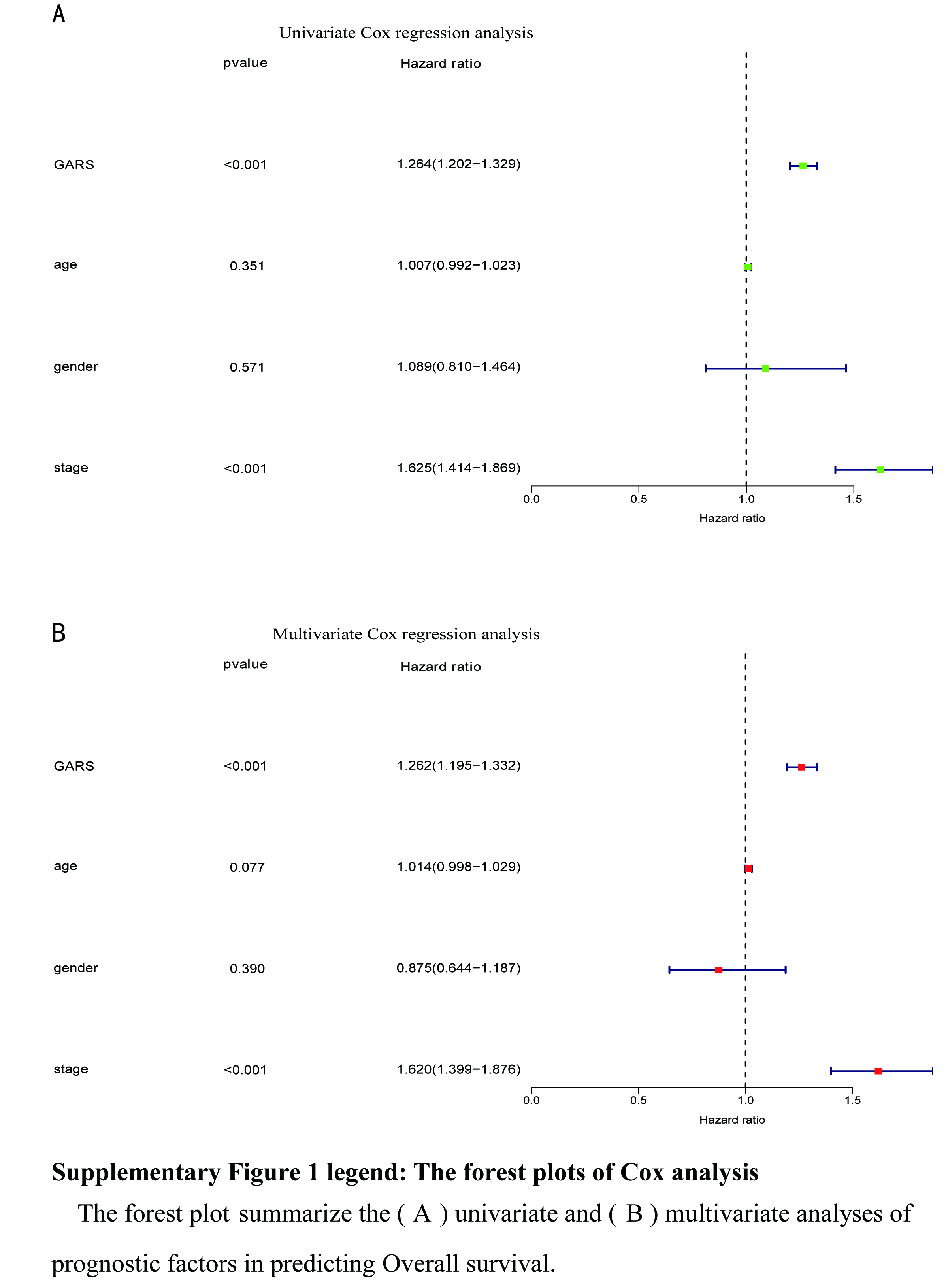

Supplement: Supplementary file 2 [file Image1.TIF]
